# OpenWorkstation: A modular open-source technology for automated *in vitro* workflows

**DOI:** 10.1016/j.ohx.2020.e00152

**Published:** 2020-10-20

**Authors:** Sebastian Eggert, Pawel Mieszczanek, Christoph Meinert, Dietmar W Hutmacher

**Affiliations:** a Centre in Regenerative Medicine, Institute of Health and Biomedical Innovation, Queensland University of Technology, Brisbane 4000, QLD, Australia; b School of Mechanical, Medical and Process Engineering, Science and Engineering Faculty, Queensland University of Technology, Brisbane 4000, QLD, Australia; c Chair of Medical Materials and Implants, Department of Mechanical Engineering and Munich School of BioEngineering, Technical University of Munich, Garching 85748, Germany; d ARC ITTC in Additive Biomanufacturing, Institute of Health and Biomedical Innovation, Queensland University of Technology, Brisbane 4000, QLD, Australia

**Keywords:** Automation, Open source hardware, Hydrogels, Modularity, 3D cell culture, Workstation, Bioprinting, Additive biomanufacturing, Reproducibility, Liquid handling robot

## Abstract

Automation liberates scientific staff from repetitive tasks, decreases the probability of human error and consequently enhances the reproducibility of lab experiments. However, the use of laboratory automation in academic laboratories is limited due to high acquisition costs and the inability to customize off-the-shelf hardware. To address these challenges, we present an Open Source Hardware concept, referred to as OpenWorkstation, to build an assembly line-inspired platform consisting of ready-to-use and customizable modules. In contrast to current standalone solutions, the OpenWorkstation concept enables the combination of single hardware modules – each with a specific set of functionalities – to a modular workstation to provide a fully automated setup. The base setup consists of a pipetting and transport module and is designed to execute basic protocol steps for *in vitro* research applications, including pipetting operations for liquids and viscous substances and transportation of cell culture vessels between the modules. We demonstrate the successful application of this concept within a case study by the development of a storage module to facilitate high-throughput studies and a photo-crosslinker module to initiate photo-induced polymerization of hydrogel solutions. We present a Systems Engineering framework for customized module development, guidance for the design and assembly of the presented modules, and operational instructions on the usage of the workstation. By combining capabilities from various open source instrumentations into a modular technology platform, the OpenWorkstation concept will facilitate efficient and reliable experimentation for *in vitro* research. Ultimately, this concept will allow academic groups to improve replicability and reproducibility in cell culture process operations towards more economical and innovative research in the future.

## Graphical abstract

**Figure:**
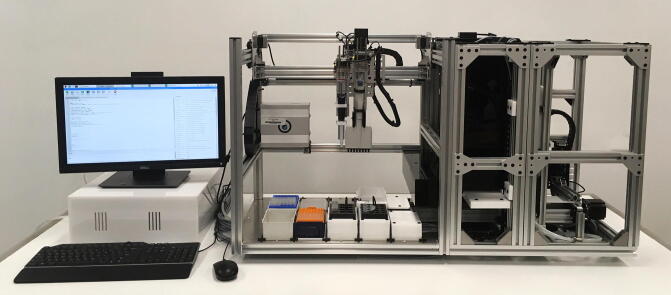

Specifications table

**Table:** 

Hardware name	OpenWorkstation: A modular open source technology for automated *in vitro* workflows
Subject area	• Engineering and Material Science • Chemistry and Biochemistry • Medical (e.g. Pharmaceutical Science) • Biological Sciences (e.g. Microbiology and Biochemistry) • Educational Tools and Open Source Alternatives to Existing Infrastructure
Hardware type	• Biological sample handling and preparation • Electrical engineering and computer science • Mechanical engineering and materials science
Open Source License	*Creative Commons Attribution-ShareAlike 4.0 International (CC BY-SA 4.0)*
Cost of Hardware	*Variable, dependent on user customization:* *Base frame with transportation module and control box: $ 900* *Module #1: pipetting module (incl. commercial product): $ 4070* *Module #2: crosslinker module: $ 675* *Module #3: storage module: $ 690*
Source File Repository	https://doi.org/10.5281/zenodo.3986643
	https://github.com/SebastianEggert/OpenWorkstation
	https://github.com/SebastianEggert/OpenWorkstation_hardware

## 1 Hardware in context

Science relies on the fundamental proficiency to reproduce and replicate research findings [1]. While reproducibility and replicability are often used interchangeably [2], the National Science Foundation defines reproducibility as ‘the ability of a researcher to duplicate the results of a prior study using the same materials and procedures as were used by the original investigator’ and replicability as ‘the ability of a researcher to duplicate the results of a prior study if the same procedures are followed but new data are collected’ [3]. Although science is built upon these two premises, global evidence on the inability to reproduce and replicate findings from high-impact biomedical journals has been growing for the last decade [2], [4], [5], [6], [7]. Multiple papers identified factors contributing to irreproducibility of *in vitro* studies, including biological variability [4], unavailable raw data from the original lab [8], low number of replicates [8], [9], biased study design [9], and lack of standardized methods [10]. Major contributing factors are simple and repetitive tasks, such as manual pipetting operations, which increase the risk of human errors and introduce non-standardized workflows [4]. Especially manual pipetting operations in complex experimental designs with increased sample throughput are fuelled with a large number of possibilities to introduce errors which manifest as inaccurate results. One practical way to reduce the number of manual tasks is the introduction of automation to reduce human error, increase efficiency, and provide increased sample throughput [11], [12], [13], [14], [15].

Although life science research, especially for industrial applications, has undergone increased automation [13], [16], most academic research groups around the world still rely on manual-based, low-throughput, time and labour intensive workflows in wet-lab experiments. Especially tasks, such as media change, generation of gradients or dilution series, and cell seeding involve rather simple and repetitive pipetting operations and do not require human intervention *per se*, but could be carried out using automated protocols [17]. Essential barriers in acquiring laboratory automation are high costs and the inability to customize off-the-shelf hardware to adapt to changing experimental requirements [18], [19].

To broaden access to cutting-edge research capabilities and democratize research, first hacker spaces followed by research groups began to design, build, and customize scientific hardware, and to freely share the details of the hardware design including the software to operate the hardware [19], [20], [21]. Such hardware, offered through free and open-source software, is considered as Open Source Hardware (OSH) [21]. Popular OSH projects, such as the Arduino platform [22] and the RepRap 3D printer [22], enable fast and inexpensive prototyping to manufacture 3D-printed parts [21], [23] to accelerate product development cycles [24]. Within the last decade, the number of OSH in scientific journals increased rapidly, ranging from liquid handling robots [25], [26], [27], [28] to a micro syringe autosampler [29], from positioning stages for automated microscopy [30], [31] to entire imaging systems [32], [33], [34], from syringe and flow pump solutions [35], [36], [37], [38] to complex microfluidic solutions [39], [40], [41], and to automated experimental platforms for biological research [42], [43].

Since most of these OSH projects present standalone solutions, they only accommodate a fixed set of functionalities. Thus, synchronized operation with other OSH or commercial open source equipment is very cumbersome. Moreover, the most OSH projects remain inflexible to adapt to changing research requirements, such as the integration of additional functionalities. Until now, an OSH concept enabling the combination of different OSH projects to automate experiments and adapt quickly to changing requirements is still missing for Life Science research in academic environments.

To address these limitations, we present an OSH assembly line concept, referred to as OpenWorkstation, to develop a modular platform consisting of customizable and ready-to-use modules. The base setup consists of a pipetting and transport module, and is designed to execute basic protocol steps for *in vitro* research applications, including pipetting operations of liquids (e.g. media, washing buffer) and viscous substances (e.g. hydrogel stock solutions) and transportation of cell culture vessels between the modules. The successful implementation of the OpenWorkstation concept is demonstrated with a case study in which we engineered a storage module to facilitate high-throughput studies and a photo-crosslinker module to initiate photo-polymerization of hydrogel solutions used for tissue engineering applications [44], [45], [46], [47]. The workstation is validated by the evaluation of the performance characteristics, such as the motorized positioning system, and the successful adoption is demonstrated by the manufacturing of various hydrogel-based 3D constructs and fully automated media change.

## 2 Hardware description

### 2.1 Systems Engineering

**Fig. 1:**
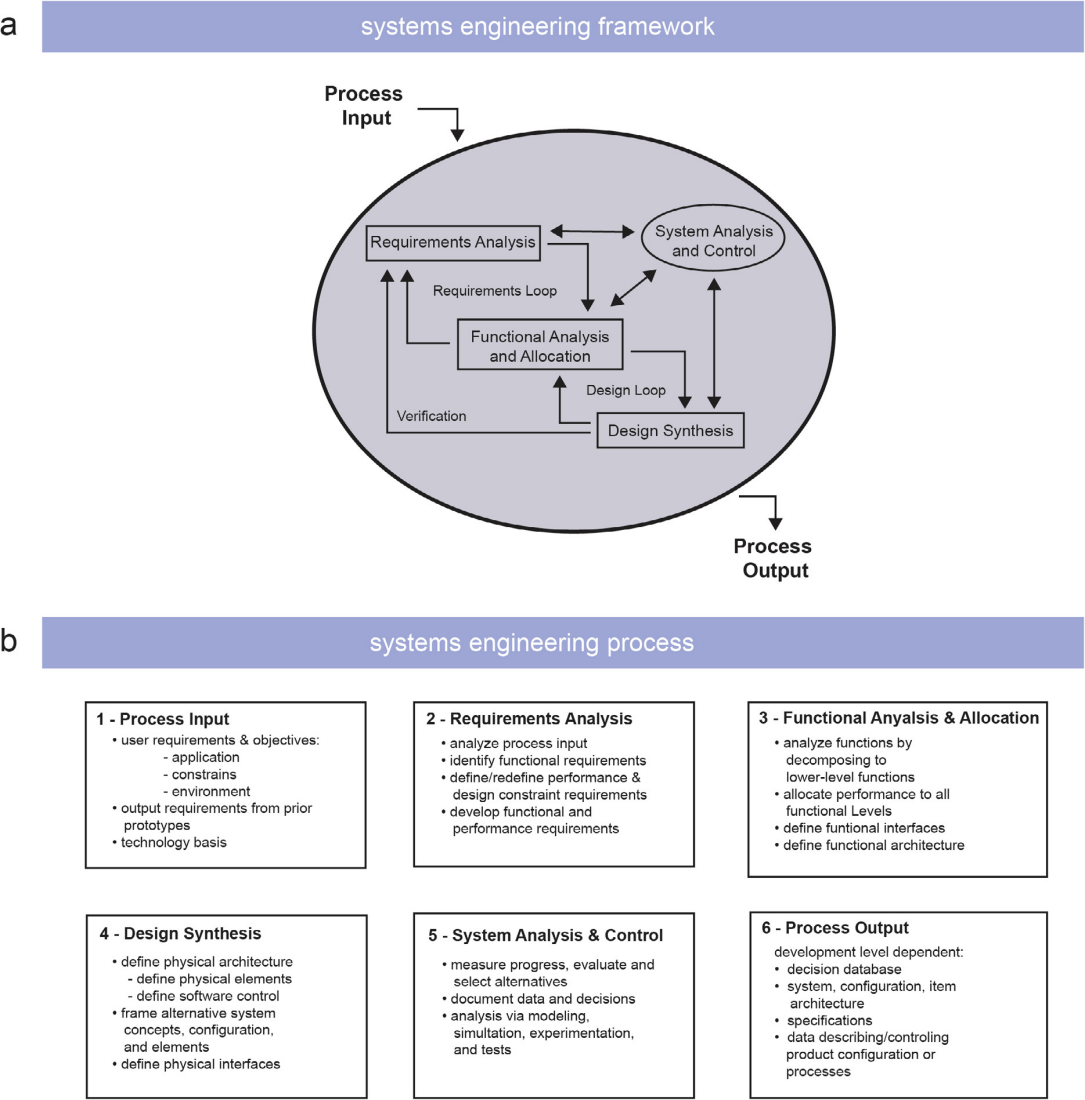
Illustration of the Systems Engineering (a) framework and (b) process. Figure a is inspired from reference [48].

To guide and support ‘the developer’ throughout the engineering process, we propose a Systems Engineering framework, a design methodology to structure, organise, and manage the degree of complexity during the engineering process (Fig. 1a, b) [48]. Systems Engineering approaches have been discussed in length in the literature [48], [49], [50], [51] and suggest the following engineering approach. First, the ‘Process Input’ is defined by specifying the user requirements, needs, and objectives including application, constraints, and environment. In addition to input requirements, process output requirements are specified to ensure that the end product meets the user requirements. Next, a ‘Requirement Analysis’ is performed to analyze the ‘Process Input’ to derive functional and performance requirements. These requirements are then analyzed in the ‘Functional Analysis and Allocation’ by decomposing higher-level functions into lower-level functions. The evaluation and results are then described as the functional architecture and allows for a more detailed understanding of the system’s requirements and functions. This analysis and allocation process leads into the ‘Design Synthesis’, where the physical (hardware) and digital (software) solutions are defined and developed. The systems engineering approach is a theoretical framework for this project and was practically applied to the presented case study in Section 2.4.

### 2.2 Concept for an assembly line inspired workstation

**Fig. 2:**
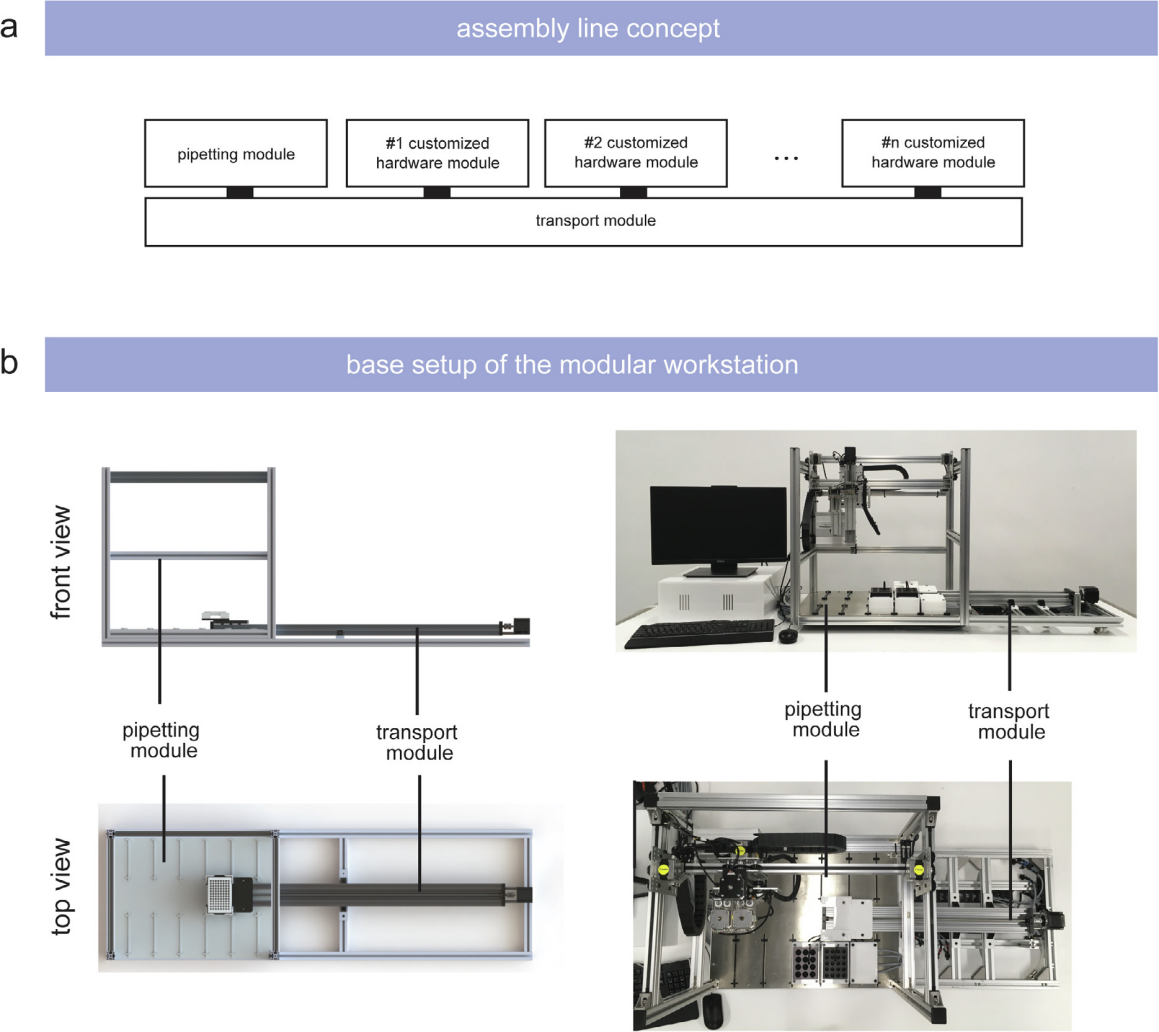
Schematic of (a) the assembly line concept and (b) the engineered base setup.

Inspired by industrial assembly lines, we aimed for an approach to engineer a workstation where samples are being transported through customizable and reconfigurable modules (Fig. 2/a). To do so, we developed (i) a modular concept, (ii) customizable hardware modules, and (iii) fully accessible application programming interface (API). We describe a setup with a modular architecture, wherein we use one module respectively to implement a set of diverse functional elements [52]. The base setup of the assembly line-inspired concept accommodates a pipetting and a transport module as essential elements (Fig. 2/b). A transport module is required to move samples between the modules to connect the implemented hardware modules with each other. A pipetting module is installed for essential pipetting operations which are required for almost every *in vitro* study. This general set up can then be customized to suit diverse experimental requirements by adding different numbers and kinds of hardware modules executing the desired functionality. Within these modules, each hardware module as well as the configuration of the hardware modules can be individually customized according to the experimental requirements. This ‘drag and drop’ approach enables a high degree of versatility, since the components as well as their configuration can be customized to fit the given requirements, including the option to remove components from the platform non-destructively without interfering with the functional operation of the other modules [53].

### 2.3 General module description

#### 2.3.1 Computational module

A computer is utilized to operate the workstation by providing an interface and sending the experimental protocol to the implemented control boards which forward commands to the functional parts of the hardware modules. A Python API has been developed to write protocol scripts to operate the entire workstation including all hardware modules in a synchronized mode. Python has been selected as the main programming language, since it is open source and a high-level and general-purpose dynamic language that focuses on code readability. Some of Python’s main advantages are (i) the extensive support libraries; (ii) the numerous third-party modules via the Python Package Index (PyPI) to enable interactions with other languages and platforms; (iii) the open source character and the community-driven development to facilitate collaborations and ease of use; and (iv) the increased productivity to build scalable multi-protocol network applications.

#### 2.3.2 Control board module

A control board translates the received protocol script into a language to operate the functional components of the hardware modules. We selected the smoothieboard (The Smoothie Project, http://smoothieware.org) as the control board module due to the range of available sizes (e.g. from 3× to 5× axis control), the numerous capabilities (e.g. up to 5× axis control with 1/32 microstepping, 6× end stops, up to 3× MOSFETs with 12.5A max, and 3× MOSFETs with 3A max, ethernet and USB connections), the 32-bit microcontroller, the available documentation, and the overall quality to price ratio. The Smoothie Project is an open source project supported by the community to design open source hardware controllers with open source firmware (https://github.com/Smoothieware). The following motion control boards can be recommended as alternatives, but have not been tested and might require additional software engineering for successful implementation: Duet3D^2^, Panucatt Azteeg X5 GT^3^, TinyG CNC Controller Board^4^.

#### 2.3.3 Hardware modules

The customized hardware modules operate a set of specific functions required to execute the experiment. The combination of functional components into one hardware module introduces an efficient and agile approach, since a single module can be easily modified without interfering with other modules. To do so, functional components are integrated into an aluminium frame to enable easy module handling and provide a robust solution for moving parts. Moreover, the various connection modes of the aluminium profiles (e.g. t-slots, nuts, threated holes, corner brackets, etc.) enable fast and inexpensive design iterations. Pre-configured aluminium frames as well as assembled mechanical parts (e.g. linear stages) are available for developers as ready-to-use assemblies to support and accelerate the design and development process. Under Section 2.4, we demonstrate the feasibility for customized hardware module development and, subsequently, successful adoption of this concept for laboratory automation in the context of a case study.

#### 2.3.4 Monitoring modules

As monitoring capability, we understand the measurement of process variables either continuously and/or repeatedly during the course of the experiment. In general, monitoring modules may include camera-based systems or various sensor types (e.g. temperature, pH, oxygen, lactate, etc.) to record the variables and document the changes over time. Implemented functions can also be used in the sense of an in-process control unit to automatically adjust operational variables via advanced process algorithms. Such in-process control units ensure consistent product quality or reach defined product output specifications.

Given the modular the flexible setup, generally any open-source microcontroller (e.g. Arduino, CircuitPython Boards), which can be operated with a Raspberry Pi, can be added to the platform. Sensors (e.g. Adafruit sensors) are either directly connected to a Raspberry Pi or an implemented microcontroller. Depending on the software integration, it is possible to operate the entire workstation including the monitoring module with one protocol script and also record parameters during the process. The presented case-study implements a Raspberry Pi camera to image photo-crosslinked hydrogels in well plates. Generally, integrated imaging processing capabilities can be easily addressed with additional python modules, such as OpenCV. For big data, high performance single board computers, such as the Google Coral Dev Board, may be required for smooth operation.

### 2.4 Case study: Customized hardware module development

**Fig. 3:**
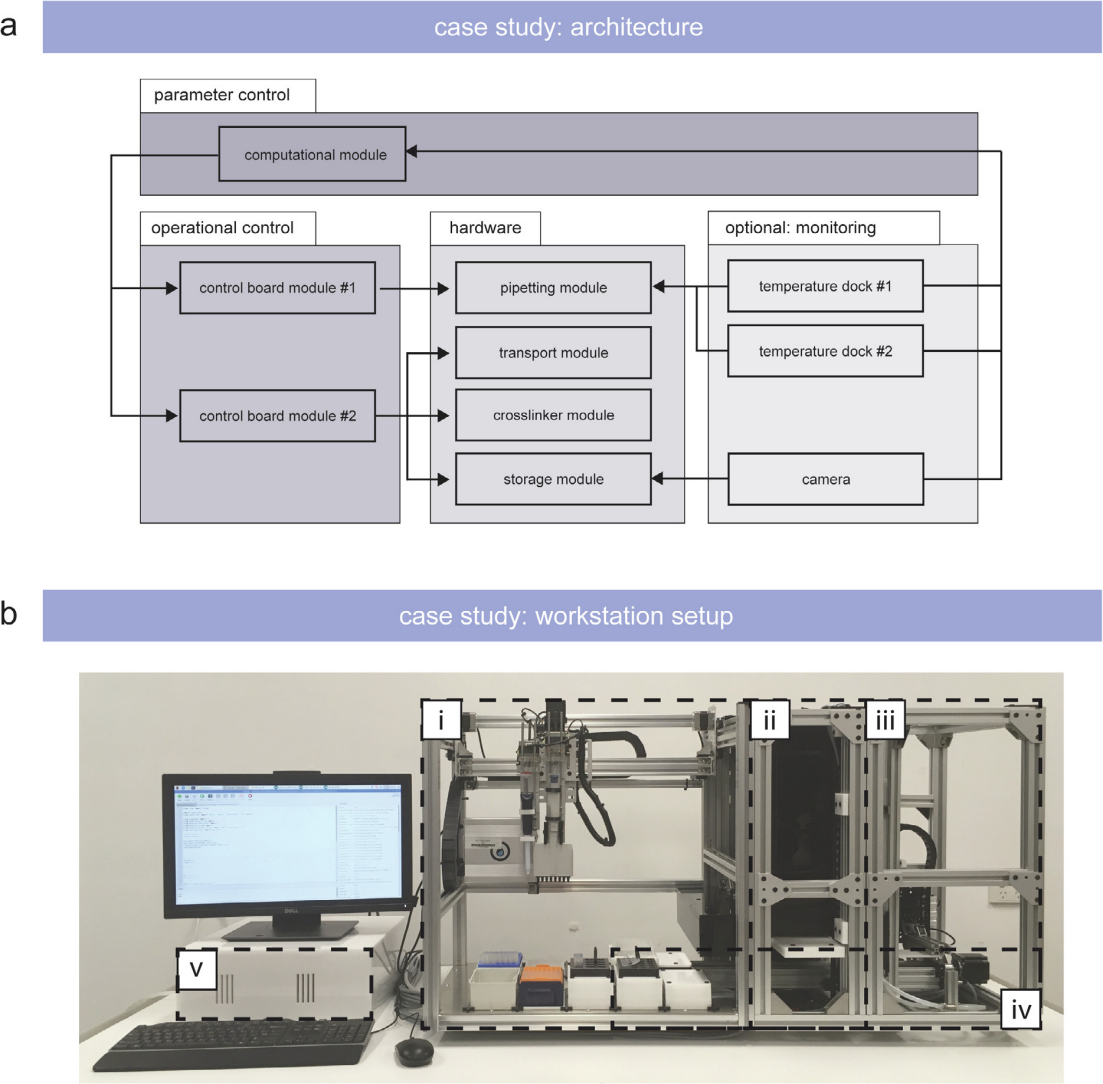
(a) Architecture and (b) setup developed for the case study. The case study describes the development and application of the following modules: (i) pipetting module, (ii) crosslinker module, (iii) storage module, (iv) transport module, and (v) computational module including control box.

A case study is presented to demonstrate the feasibility for customized hardware module development and, subsequently, successful adoption of the OpenWorkstation concept for laboratory automation (Fig. 3/a, b). The overall objective of the case study was to automate 2D and 3D cell culture workflows by the development of specialized hardware modules. As current handling tasks for 2D and 3D cell culture are mostly manual-based in a low-throughput in an academic setting, we aimed to move from a manual-based process to an automated workflow. Within this study, we focused on the usage of hydrogels to enable the manufacturing of physiologically relevant 3D microenvironment [44], [45], [46]. Hydrogels are water-swollen 3D polymer networks which are structurally maintained by chemical and/or physical crosslinking of polymer chains [44].

To understand the need for automated workflows for 2D and 3D cell culture, it is essential to comprehend the basic handling tasks. Current steps rely on repetitive and time-consuming tasks which are mostly executed manually in low throughput fashion. The current workflow for 3D cell culture tasks is summarized in Supplementary Fig. 1 and includes various manual mixing and distributing steps, including the dilution of the stock solution, mixing with a photoinitiator system, addition und subsequent mixing with cells, distribution into the well plates, crosslinking, and manual transportation of plates. The associated limitations of this workflow are discussed in detail in a recent perspective article [17].

**Fig. 4:**
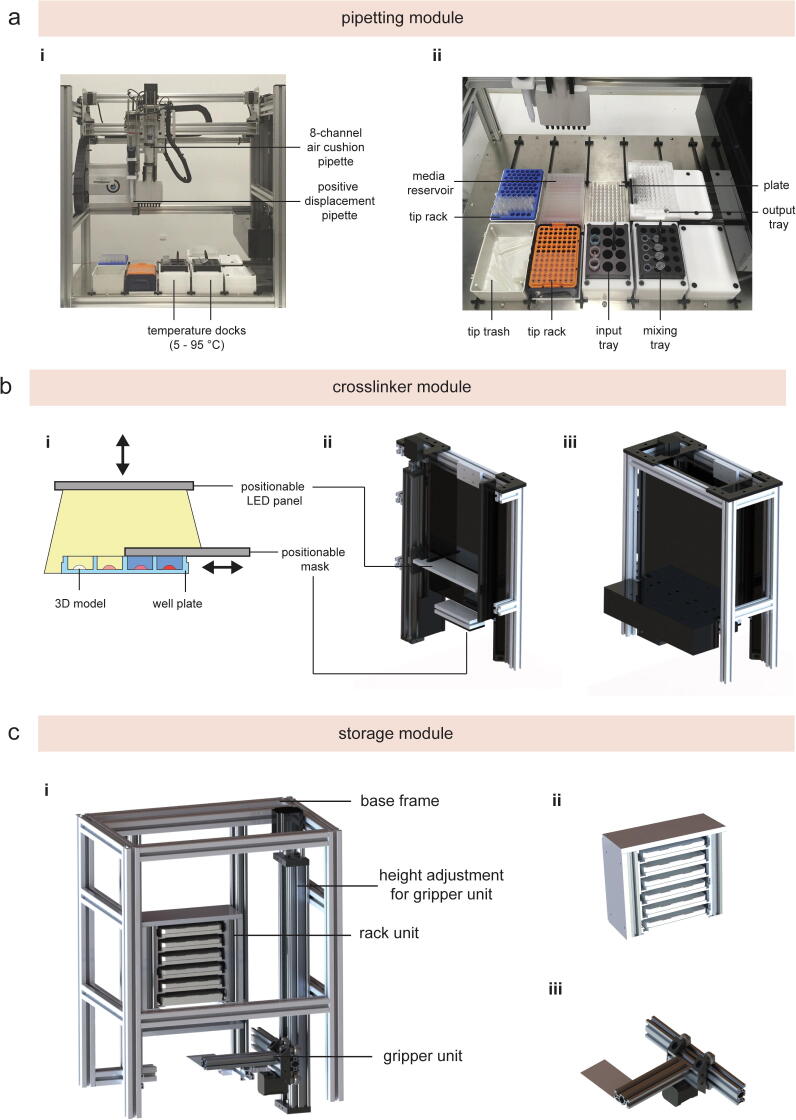
Images of the (a) pipetting module and rendered images of (b) the photo-crosslinker and (c) storage module.

Based on the overall objective of the case study, we applied a Systems Engineering framework and defined the following process input for the case study: Automation of current 2D and 3D cell culture workflows by the development of specialized hardware modules. This process input was applied to specify the following requirements used for the subsequent functional analysis and allocation, and the final design synthesis. Due to the handling of liquids (e.g. media, washing buffer, etc.) and viscous substances (e.g. glycerol, hydrogel stock solutions, etc.), aspirating and dispensing capabilities are required to transfer defined volumes for preparation, mixing, and 3D model manufacturing tasks. Positive displacement solutions ensure accurate aspirating and dispensing volumes for viscous liquids and temperature adjustment for samples enable the processing of thermoresponsive materials. This functional analysis & allocation leads to the development of a pipetting module equipped with positive-displacement pipettes and temperature dock(s) within the design synthesis (Fig. 4/a). Chemical crosslinking is required to initiate photo-induced polymerization and was realized by a separate crosslinker module with an exchangeable panel equipped with LEDs with a defined wavelength and addressable intensity (Fig. 4/b). Light intensity (e.g. mW/cm^2^) can be adjusted accordingly in the protocol script. To enable the generation of defined exposure gradients (e.g. from 1 to 12 min with 1 min steps), a sliding mask was integrated into the crosslinker module. A further specified requirement included the execution of automated high-throughput studies. The ability to increase the sample number and enable a fully automated workflow was realized by a separate storage module (Fig. 4/c). This storage module includes exchangeable rack units accommodating six well plates and integrates a lid lifting function to open or close the cell culture vessels without the need for human interference. Since the workstation is intended to be used for cell studies, enclosed and ventilated laboratory workspace is compulsory to avoid sample and environmental contamination, and, therefore, the footprint is designed to fit into an off-the-self Class II biological safety cabinet.

The presented case study is built upon the assembly line concept, and the engineered workstation consists of the base setup (pipetting and transport module) as well as the two customized modules (crosslinker and storage module) (Fig. 3/b):

**Transportation module:** sample movement between the modules
**Pipetting module:** dispensing and aspirating tasks
**Photo-crosslinker module:** photo-induced crosslinking for hydrogels
**Storage module:** increased sample throughput

### 2.5 Advantages of a modular open-source workstation

The open-source character is a key feature to empower scientists, enable community-driven development and provide affordable solutions, and additional literature on potential of Open Source Hardware is available [19], [21], [39], [54], [55], [56], [57]. However, most Open Source Hardware projects are standalone solutions; thus presenting only one solution with a specific functionality. Although additional functionalities may be added, the system is not designed in a way which makes it easy to change quickly to new requirements. In addition, physical connection and synchronised operation with other open-source projects or commercial equipment is very cumbersome. To address this limitation, we introduce the OpenWorkstation concept which is a modular approach to allow simple and fast module integration and removal. Firstly, a modular approach enables module integration and removal without disassembling the entire system, since the dependencies between the module are kept at a minimum. This allows easy development and integration of new modules to adapt the system to changing requirements. By integrating new functionalities, the system can be quickly upgraded and thus allows the system to evolve over the years. Since single modules are designed and engineered separately, the complexity in the development phase is reduced. If issues arise with a specific module, the module can be easily removed and exchanged without having an impact on other modules. Overall, the combination of an open-source features with a modular approach makes it possible to adapt the system cost-effectively and quickly to changing requirements.

## 3 Design files

The following design (Table 1) files are necessary to complete this build and are available in the Zenodo repository in editable file formats.

**Table 1:** Design file summary.

Design file name	File type	Open source license	Location of the file
Build instructions	*PDF*	CC BY-SA 4.0	Available with the article, Zenodo: https://doi.org/10.5281/zenodo.3986643
Bill of materials (BOM), detailed with links	*XLSX*	CC BY-SA 4.0	Available with the article, Zenodo: https://doi.org/10.5281/zenodo.3986643
Design files	*CAD*	CC BY-SA 4.0	Zenodo: https://doi.org/10.5281/zenodo.3986643
Laser cut parts	*CAD*	CC BY-SA 4.0	Zenodo: https://doi.org/10.5281/zenodo.3986643
Pin configuration for smoothieboard	*PDF, AI*	CC BY-SA 4.0	Zenodo: https://doi.org/10.5281/zenodo.3986643
Electronic circuits	PDF	CC BY-SA 4.0	Available with the article, Zenodo: https://doi.org/10.5281/zenodo.3986643
Software	Python code	CC BY-SA 4.0	Zenodo: https://doi.org/10.5281/zenodo.3986643
			GitHub: https://github.com/SebastianEggert/OpenWorkstation

### 3.1 Computer-aided design (CAD) files

This section includes CAD files for the developed crosslinker, storage, and transportation modules as well as the frames used as for basic hardware module design. All CAD files are available as SolidWorks files.

**workstation.sldasm** – CAD assembly including all single components
**moduleFrame_overview.sldasm** – overview of frame geometries

### 3.2 Laser-cut parts

Custom designed parts are required for module assembly and are manufactured from acrylic plates using a laser cutter. All custom parts are saved as CAD files. Custom parts can be cut in-house or can be ordered from local plastic suppliers/3rd party. Alternatively, parts can be printed using 3D printers.

### 3.3 Build instructions

**build-instructions_v0.1.pdf** – build instructions for each module

### 3.4 Bill of materials

**workstation_bom_v0.1.xlsx** – multi-sheet part list for each developed module including cutting list for aluminium profiles, if additional cutting is required

### 3.5 Pin configuration for smoothieboard

**workstation_pin-configuration_v0.1.pdf**– pin configuration for smoothieboard v1.1 used in the case study

### 3.6 Electronic circuits

**workstation_electrical-circuit_v0.1.pdf** – electronic circuits as used in the case study

### 3.7 Open source software and firmware

**workstation API** – A Python framework was designed to write protocol script and operate the functional elements of the modules. A README file and all code dependencies are included in the repository unless noted. Example files are included in the repository.
**Opentrons API** – A Python framework was designed to write protocol scripts for the pipetting module (OT-1).
**Firmware** – Firmware update for smoothieboard to connect with the implemented APIs.

## 4 Bill of materials

A list of materials to be purchased for the presented workstation can be found as an Excel spreadsheet in the Supplementary Materials and in the GitHub repository. The bill of materials (BOM) is divided into materials required for the computational module, control box including electronics, frame, transportation module, pipetting module, crosslinker module, storage module (Table 2).

**Table 2:** Bill of Materials. Parts are purchased by module. Any “0″ quantity denotes that this part has been previously purchased in the electronics section in a sufficient number.

Component	Source	Catalog Number	Quantity	Cost/Unit (USD)	Total Cost (USD)
Computation module					
Raspberry Pi 3 Model B+	Adafruit Industries (USA)	3775	1	35.00	35.00
Raspberry Pi Power Supply	Adafruit Industries (USA)	1995	1	7.50	7.50
MicroSD card with NOOBS	Adafruit Industries (USA)	3259	1	9.95	9.95
Arduino Mega	SparkFun Electronics, Inc (USA)	DEV-11061	1	38.95	38.95
MOSFET-FQP30N06L	SparkFun Electronics, Inc (USA)	COM-10213	2	0.95	1.90
Breadboard, 170 pin (max 5A rating)	Newark element14 (USA)	71Y9231	2	1.53	3.06

Control box and electronics					
Smoothieboard, 5XC-R, version1.1, voltage reg 1A	Uberclock, LL (USA)	smoothie_5x	1	189.00	189.00
Fan, 80 mm, 12 V	Newark element14 (USA)	29 M8108	2	11.39	22.78
Fan guard for 80×80 mm fan	Newark element14 (USA)	56P2990	4	1.07	4.28
power supply 12 V/29A (Meanwell) set	OpenBuilds (USA)	511-Set	1	59.99	59.99
2-core cable, screened, 0.5 mm^2^	Newark element14 (USA)	50AC7152	1	26.90	26.90
4-core cable, screened, 0.5 mm^2^	Newark element14 (USA)	50AC7160	1	50.60	50.60
Xtension connectors 2pin	OpenBuilds (USA)	2525-Set	6	1.49	8.94
Xtension connectors 4pin	OpenBuilds (USA)	2525-Set	5	3.49	17.45
Acrylic plates 10 mm, black	Mulford Plastics Pty Ltd (Australia)	ASTARIGLAS-GP-10 mm-black	1.5 m^2^	99.90	99.90
Acrylic plates 10 mm, white	Mulford Plastics Pty Ltd (Australia)	ASTARIGLAS-GP-10 mm-white	1.5 m^2^	99.90	99.90
Acrylic plates 6 mm, white	Mulford Plastics Pty Ltd (Australia)	ASTARIGLAS-GP-6 mm-white	0.5 m^2^	19.90	19.90

Frame					
Profile 30 × 30 (slot 8 mm)	GAP Engineering Pty Lty (Australia)	084.107.002	4.84 m	10.50	50.82
Clamping angle 25 × 40	GAP Engineering Pty Lty (Australia)	084.305.002	12	2.45	29.40
Nut M8 – slot 8 (pack of 25)	GAP Engineering Pty Lty (Australia)	084.302.037	1	17.00	17.00
Bolt M8 × 16 mm – slot 8 (pack of 25)	GAP Engineering Pty Lty (Australia)	084.301.002	1	4.00	4.00
Drag chain cable carrier: 1 m	OpenBuilds (USA)	2450	2	15.99	47.97
cable drag chain mounting bracket	Makerstore (Australia)	BRAC-CDC	6	3.70	22.00

Transport module					
1000 mm C-Beam® XLarge Linear Actuator Bundle (w NEMA23)	OpenBuilds (USA)	2495-Bundle	1	174.99	174.99
Xtension Limit Switch Kit	OpenBuilds (USA)	2805-Kit	1	4.99	4.99
Nut M5 – slot 8 (pack of 25)	GAP Engineering Pty Lty (Australia)	084.302.002	1	3.55	3.55
Tee nuts (M5) (pack of 10)	OpenBuilds (USA)	536-Pack	1	2.99	2.99
M5 × 15 mm (pack of 10)	OpenBuilds (USA)	922-pack	2	1.59	3.18
Black corner brackets	OpenBuilds (USA)	540	8	2.99	23.92
Drag chain cable carrier (10 × 15 mm): 1 m	OpenBuilds (USA)	2450	3	15.99	47.97

Pipetting module					
OT-One S Hood	Opentrons Laboratories Inc (USA)	OT-One S Hood	1	4,000.00	4000.00
Waterproof DS18B20 Digital temperature sensor	Adafruit Industries (USA)	381	2	9.95	19.90
Peltier thermo-electric cooler module (12 V, 5A)	Adafruit Industries (USA)	1330	4	11.95	47.80
2-core cable, screened, 0.5 mm^2^	Newark element14 (USA)	50AC7152	0	26.90	0.00
4-core cable, screened, 0.5 mm^2^	Newark element14 (USA)	50AC7160	0	50.60	0.00
xtension connectors 2pin	OpenBuilds (USA)	2525-Set	0	1.49	0.00
Xtension connectors 4pin	OpenBuilds (USA)	2525-Set	0	3.49	0.00
Acrylic plates 10 mm, white	Mulford Plastics Pty Ltd (Australia)	ASTARIGLAS-GP-10 mm-white	0		0.00

Crosslinker module					
500 mm C-Beam® Linear Actuator Bundle (w NEMA23)	OpenBuilds (USA)	995-Bundle	1	144.99	144.99
250 mm C-Beam® Linear Actuator Bundle (w NEMA23)	OpenBuilds (USA)	995-Bundle	1	126.99	126.99
Xtension Limit Switch Kit	OpenBuilds (USA)	2805-Kit	2	4.99	9.98
Profile 30 × 30 (slot 8 mm)	GAP Engineering Pty Lty (Australia)	084.107.002	4.26 m	10.50	44.73
Profile 20 × 20 (V slot): 500 mm	OpenBuilds (USA)	VSlot20x20LinearRail	3	5.49	16.47
Clamping angle 25 × 40	GAP Engineering Pty Lty (Australia)	084.305.002	4	2.45	9.80
Nut M5 – slot 8 (pack of 25)	GAP Engineering Pty Lty (Australia)	084.302.002	1	3.55	3.55
Bolt M5 × 16 mm – slot 8 (pack of 25)	GAP Engineering Pty Lty (Australia)	084.301.009	1	2.88	2.88
Black corner brackets	OpenBuilds (USA)	540	12	2.99	35.88
Tee nuts (M5) (pack of 10)	OpenBuilds (USA)	536-Pack	3	2.99	8.97
M5 × 8 mm (pack of 10)	OpenBuilds (USA)	946-pack	3	1.39	4.17
M5 × 10 mm (pack of 10)	OpenBuilds (USA)	878-pack	3	1.49	4.47
M5 × 15 mm (pack of 10)	OpenBuilds (USA)	922-pack	3	1.59	4.77
M5 × 20 mm (pack of 10)	OpenBuilds (USA)	750-pack	3	1.69	5.07
90°joining plate	OpenBuilds (USA)	610	8	4.99	39.92
Aluminium spacer: 6 mm	OpenBuilds (USA)	90-Pack	4	2.99	11.96
LED strip light: 399 nm (reel, 5 m)	Flexfire LEDs, Inc.(CA, USA)	CB-UV-12 V-16FT	1	175.00	175.00
Drag chain cable carrier (10 × 15 mm): 0.5 m	OpenBuilds (USA)	2450	3	7.99	23.97
2-core cable, screened, 0.5 mm^2^	Newark element14 (IL, USA)	50AC7152	0	26.90	0.00
4-core cable, screened, 0.5 mm^2^	Newark element14 (IL, USA)	50AC7160	0	50.60	0.00
Xtension connectors 2pin	OpenBuilds (USA)	2525-Set	0	1.49	0.00
Xtension connectors 4pin	OpenBuilds (USA)	2525-Set	0	3.49	0.00
Acrylic plates 10 mm, black	Mulford Plastics Pty Ltd (Australia)	ASTARIGLAS-GP-10 mm-black	0		0.00

Storage module					
500 mm C-Beam® Linear Actuator Bundle (w NEMA23)	OpenBuilds (USA)	995-Bundle	1	144.99	144.99
250 mm Belt & Pinion Actuator Bundle (w NEMA23)	OpenBuilds (USA)	2565-Bundle	1	109.99	109.99
Xtension Limit Switch Kit	OpenBuilds (USA)	2805-Kit	2	4.99	9.98
Profile 30 × 30 (slot 8 mm)	GAP Engineering Pty Lty (Australia)	084.107.002	5.3 m	10.50	0.00
Profile 20 × 20 (V slot): 500 mm	OpenBuilds (USA)	VSlot20x20LinearRail	4	5.49	21.96
Profile 20 × 20 (V slot): 250 mm (to be cut)	OpenBuilds (USA)	VSlot20x20LinearRail	8	3.29	26.32
Profile 20 × 40 (V slot): 250 mm (to be cut)	OpenBuilds (USA)	VSlot20x40LinearRail	1	3.99	3.99
Clamping angle 25 × 40	GAP Engineering Pty Lty (Australia)	084.305.002	4	2.45	9.80
Nut M5 – slot 8 (pack of 25)	GAP Engineering Pty Lty (Australia)	084.302.002	1	3.55	3.55
Bolt M5 × 16 mm – slot 8 (pack of 25)	GAP Engineering Pty Lty (Australia)	084.301.009	1	2.88	2.88
Tee nuts (M5) (pack of 10)	OpenBuilds (USA)	536-Pack	3	2.99	8.97
black corner brackets	OpenBuilds (USA)	540	12	2.99	35.88
M5 × 8 mm (pack of 10)	OpenBuilds (USA)	946-pack	3	1.39	4.17
M5 × 10 mm (pack of 10)	OpenBuilds (USA)	878-pack	3	1.49	4.47
M5 × 15 mm (pack of 10)	OpenBuilds (USA)	922-pack	3	1.59	4.77
M5 × 20 mm (pack of 10)	OpenBuilds (USA)	750-pack	3	1.69	5.07
M5 × 25 mm (pack of 10)	OpenBuilds (USA)	20-pack	1	1.79	1.79
90°joining plate	OpenBuilds (USA)	610	12	4.99	59.88
Aluminium spacer: 20 mm	OpenBuilds (USA)	65-Pack	4	2.99	11.96
Vacuum ejector	Schmalz (Germany)	10.02.01.00565	1	38.62	38.62
Bellows suction cups (oval): SPOB1 60 × 20	Schmalz (Germany)	10.01.06.03511	2	37.50	75.00
Hose, VSL 8–6 PU, external diameter D = 8 mm, internal diameter d = 6 mm	Schmalz (Germany)	10.07.09.00003		4.76	0.00
Screw plug connection, straight	Schmalz (Germany)	10.08.02.00206	2	4.47	8.94
t-Connection	Schmalz (Germany)	10.09.02.00022	1	16.43	16.43
3/2 pneumatic control valve solenoid, G1/8, 12 V	RS Components Pty Lty (Australia)	797–5093	1	42.00	42.00
Pneumatic straight threaded-to-tube adapter, G 1/8 male, push In 6 mm	RS Components Pty Lty (Australia)	812–049	2	4.20	8.40
Drag chain cable carrier (10 × 15 mm): 0.5 m	OpenBuilds (USA)	2450	3	7.99	23.97
2-Core cable, screened, 0.5 mm^2^	Newark element14 (USA)	50AC7152	0	26.90	0.00
4-Core cable, screened, 0.5 mm^2^	Newark element14 (USA)	50AC7160	0	50.60	0.00
Xtension connectors 2pin	OpenBuilds (USA)	2525-Set	0	1.49	0.00
Xtension connectors 4pin	OpenBuilds (USA)	2525-Set	0	3.49	0.00
Acrylic plates 10 mm, white	Mulford Plastics Pty Ltd (Australia)	ASTARIGLAS-GP-10 mm-white	0		0.00
Acrylic plates 6 mm, white	Mulford Plastics Pty Ltd (Australia)	ASTARIGLAS-GP-6 mm-white	0		0.00

## 5 Assembly instructions

The following build instructions are for the presented case study and include a general build description including the rationale behind the design and engineering process for the two essential modules for the base setup (pipetting module, transport module) and the two customized modules for the case study (photo-crosslinker module, storage module). The build is divided into three steps, which are (1) assembly of modules and base, (2) setup of control box including electrical assembly, and (3) workstation setup. All sections include a brief header to inform about the build duration, materials, separate equipment/tools, and provide advice on critical and cautious steps. A step-by-step building instruction is uploaded as SUPPLEMENTARY MATERIAL and will be updated on the GitHub repository. The end result will be a workstation with four hardware modules and a control box that is ready to use.

Basic mechanical and electrical engineering skills are required to setup the system. Where specific skills are necessary (e.g. soldering) additional support is provided in the form of external links. A general list of the basic tools required to assembly the system is provided in the detailed build instructions in the Supplementary Materials. We suggest that the total setup time would be around two months; however this depends highly on the mechanical and electrical skills.

### 5.1 Assembly of modules

**Timing** 4–6 weeks (depending on experience)
**Materials** linear stages, 20 × 20 mm and 30 × 30 mm aluminium profiles, screws (M3, M5, M6), clamping angles, tee nuts for M5 and M6, NEMA23 stepper motors, cable housing, laser-cut parts (quantity is specified in BOM)
**Equipment** M3, M5, M6 Allen keys
**Critical** cutting surface of aluminium profiles should be straight (90°) to ensure proper connection of end mounts and additional profiles

#### 5.1.1 Linear stages

Linear stages were designed following the ‘openbuilds’ approach and assembled according to video instructions provided online by OpenBuilds^5^^,^^6^^,^^7^. OpenBuilds provides a well-documented instruction and assembly support for the implemented linear stages. Linear stages were used as described by OpenBuilds and not further modified. Depending on the length of the transportation module, cutting of the aluminium profile might be required to adjust the overall length. Cutting of aluminium profiles can be executed in-house using a chop saw or by the supplier. As explained in the video instructions, the linear stage assembly also accommodates connection of the Nema23 stepper motor, which are then connected to a pluggable 4-pin connector.

#### 5.1.2 Base

Aluminium profiles were used to provide a mechanical base frame for structural support. Depending on the support (e.g. table, lab bench, biological safety cabinet, etc.), appropriate solutions have to be selected to provide a robust connection between the workstation and the support. If the workstation is to be placed in a Class II biological safety cabinet, the internal dimensions have to be kept in mind in the requirement analysis steps.

In the presented case study, two aluminium profiles were chosen as support and connected to a grid base structure, consisting of aluminium profiles and plate spacers. These plate spacers were used to adjust the height of the transportation module which accommodates the samples. Hence, the overall sample height to hardware modules can be adjusted to the required specifications using these plate spacers.

#### 5.1.3 Transportation module

This module transports the sample(s) to the customized modules using a linear stage and a sample holder. Depending on the overall length of the workstation, the length of the transportation module has to be selected to ensure that the sample can be transported to each module. The sample holder design can be optimized to the required experimental applications.

In this report, a standardized well plate design was selected to allow the transportation of well plates for biological samples. Starting with the assembly of the linear stage according to the provided instructions (see Section 5.1.1), customized parts for the sample holder have to be laser cut and then connected to threated holes of the gantry plate to provide a securing function for the samples.

#### 5.1.4 Pipetting module

This module adds aspirating, dispensing, and mixing capabilities for liquids and viscous substances to the workstation. For a time-efficient implementation, an open source liquid handling robot was optimized with custom-made solutions to include positive-displacement pipettes. These pipettes use capillary pistons to apply mechanical forces via a solid piston to push viscous material out of the tip, enabling reliable aspirating and dispensing tasks for liquids and viscous substances. For example, the tissue engineering and regenerative medicine (TE&RM) community utilizes hydrogels to engineer physiologically relevant 3D microenvironments for cells [44]. Up to now, these workflows could not be automated due to lack of automated pipetting solutions for these, often highly viscous, hydrogel solutions, and, therefore, hydrogels are still mainly pipetted manually [17]. By integrating positive-displacement pipettes, the pipetting module facilitates automated aspirating, dispensing, and mixing tasks for these hydrogels. Moreover, this module enables the usage of two pipettes (single- or 8-channel pipette) and offers a 8-deck capacity according to ANSI/SLAS standards (ANSI: American National Standards Institute, SLAS: Society for Laboratory Automation and Screening). Temperature docks were developed and added to the pipetting module to cool or heat samples to a defined temperature. One temperature dock consists of two Peltier elements connected to a 60 mm × 80 mm aluminium heatsink and located in a customized acrylic plater holder to fit aluminium blocks (Ratek Instruments Pty Ltd, Australia) (dimensions: L95mm × W75 × H50mm).

#### 5.1.5 Photo-crosslinker module

A crosslinker module was developed to provide photo-induced crosslinking and to enable the generation of time gradients on a well plate. To do so, the crosslinker module was designed consisting of a LED panel, a slidable mask, and a dedicated housing. The LED panel is attached to an exchangeable LED holder, which allows changing of the LED panel according to the required wavelength. The light intensity can be either adjusted by varying the height of the LED panel or via pulse width modulation (PWM). PWM is a method of reducing the supplied power to the connected device. By lowering the PWM values, less power is supplied to the LEDs and, therefore, the LED’s intensity is reduced by the specified PWM value. This method is widely adopted for 3D printers to control the fan speed. The slidable mask is operated by a linear mask and can cover parts of the well plate to create exposure gradients within one well plate by varying the exposure time.

#### 5.1.6 Storage module

A storage module has been designed and developed to increase sample throughput by providing access to six well plates kept in a rack unit within this module. Compared to stand-alone liquid handling robots [25], [26], [58], this module allows the processing of multiple well plates for increased throughput applications. Plates are stored within a rack and are positioned onto the transport module by a gripper unit (Supplementary Video 1). Two vacuum suction cups are installed under the rack unit to lift the lid of well plates.

### 5.2 Setup of control box including electrical assembly

**Timing** 1 week
**Materials** 2 and 4 core cable, solder, solder wick, heat shrink, ferrules crimper, laser cut plates (quantity is specified in BOM)
**Equipment** fine (or flat tip) soldering iron, tweezers, optical microscope to inspect soldered connections, wire cutters, wire strippers, heat gun, multimeter
**Caution** use approved or provided ferrules, since ferrule failure can result in wire creeping causing loose wires which may create short circuits
**Critical** before starting with connecting the control board(s), familiarize yourself with the pin configuration of the smoothieboard (Supplement Fig. 2) and the general electrical circuit (Supplement Fig. 3) (also available and updated on GitHub); check with the Health and Safety department, if electrical work can be executed without a license or if qualified industrial electricians are required to connect the components

The main objective of this section is to provide a summary of the electrical work required to connect the stepper motors and end stops as well as to wire the smoothieboard and Arduino. For beginners, introductions to electricity^8^, soldering^9^ and wiring^10^^,^^11^ are recommended before starting with the electrical assembly work. A dedicated control box was designed to comply with the requirements of the university’s Health and Safety guidelines. The main power circuit is a 12 V circuit, which is considered as a low-voltage application in most countries. The number of the required components (e.g. control board) is selected based on the number of hardware modules and additional functionalities (e.g. valve). It is recommended to install at least one fan to provide cooling for the electrical components.

### 5.3 Workstation setup

**Timing** under 1 h
**Materials** assembled modules and base, screws (M5, M6), clamping angles, tee nuts for M5 and M6
**Equipment** M5, M6 Allen key

After setting up the modules and the control box, the workstation can be assembled with the developed hardware modules (Fig. 3/b). For the presented case, first the pipetting and then the crosslinker and storage module are positioned next to each other. Each module is connected to the base frame by four clamping angles and also connected to the module(s) next to it. Modules are connected to the control board via the pluggable connectors to allow a plug and play scenario, if single modules need to be removed for modification (Supplementary Video 2).

### 5.4 Functional testing

After successful mechanical and electrical assembly, it is recommended to check the operation of each functional part. To do so, each implemented function should be tested briefly to identify if all cables are connected and soldered properly, and if the uploaded configuration (e.g. step size for stepper motor) is correct. Troubleshooting at this stage is easier and saves time compared to a later stage, such as fully assembled workstation. Simple functional testing can include powering the control board, operation of the stepper motor, operation of the linear stage, or ON/OFF switching of LEDs. More extensive testing is only possible after setting up the computational module and the API (see next chapter).

## 6 Operation instructions

### 6.1 Set-up of computational module

**Timing** 3–6 h
**Equipment** Raspberry Pi
**Critical** Ensure that you have at least Python 3.5 installed on your system; if not, install the latest version^12^, define and update Python path, and set Python 3.5 + as default for terminal commands

The following instructions are written for a Raspberry Pi (RPi) single-board computer; however, also Windows 7,10 and macOS 10.12 + have been successfully used with the presented workstation:

| Install Raspbian which is a Debian-based computer operating system for Raspberry Pi^13^
| Start Raspberry Pi, finish setup^14^, and connect to the internet^15^
| Open a terminal window from the taskbar or application menu and update the software using the APT (Advanced Packaging Tool) tool in a terminal:

Update the system's package list

sudo apt-get update

Upgrade all the installed packages

sudo apt-get dist-upgrade

Restart the Raspberry Pi

sudo reboot

| Install python pip^16^

sudo apt-get install python3-pip

| Create a Python virtual environment^17^

A virtual environment will prevent any interference of newly installed packages with already installed packages. By creating isolated environments, the dependencies required for different projects are kept separate.

Install the virtualenv tool

pip install virtualenv

Decide upon a directory where you want to place it the virtual environment ‘OpenWorkstation’

python -m venv OpenWorkstation

This will create the OpenWorkstation directory and also create directories inside it containing a copy of the Python interpreter, the standard library, and various Supporting Files.^18^

Activating the virtual environment

source OpenWorkstation/bin/activate

| Install openworkstation API

pip install openworkstation

| Install opentrons API for OT-1^19^

pip install opentrons==2.5.2

| Test successful API installation

python3

>>> import openworkstation

>>> import opentrons

| Clone GitHub project to access CAD models and protocol scripts

git clone https://github.com/SebastianEggert/OpenWorkstation.git

### 6.2 Update configuration file on smoothieboard

The configuration of the connected peripherals (e.g. stepper motor) is stored on the smoothieboard's SD card and has to be updated initially. When parts with a different specification (e.g. step size of a stepper motor) are used, the configuration files have to be modified to accommodate the physical changes. A list of most configuration options is provided by the Smoothie Project^20^. The configuration for the developed modules with the implemented parts can be found in the design files section.

### 6.3 Operation via Python script

In order to start operating the entire workstation or each module, the terminal function or a Python integrated development environment (IDE) (e.g. Thonny^21^, IDLE^22^) is used to send the Python script from the computer to the control board. For the pipetting module, detailed operational instruction for the OT-1 is provided by Opentrons^23^. Detailed instructions for the workstation API commands are found in the README file and on the GitHub repository. Example files are included to provide additional guidance on the generation of protocols.

If new hardware modules are being developed, a separate ‘module*Name*.py’ file is being added with the hardware module specific commands. Afterwards, the ‘protocol.py’ file needs to be revised to import the new ‘module*Name*.py’ file with the custom commands. The following section provides a description about setting up and using the software.

#### 6.3.1 Import

Always include the OpenWorkstation API within your file:

import openworkstation

Currently, specific commands for a single module are saved in a separate module file. For example, the positions for the transport module are saved in the ‘moduleTransport.py’ file. These module-specific have to be imported into the main ‘protocol.py’ file. General commands, such as connect to USB port and homing, are defined in a separate ‘commands.py’ file. Example files are available in the GitHub repository.

from commands import *

from modulePipetting import *

from moduleCrosslinker import *

from moduleStorage import *

from moduleTransportation import *

#### 6.3.2 Connect to USB port(s)

The command ‘connect()’ will connect to board to the USB port(s) which are defined in the ‘commands.py’ file:

robot2USB = '/dev/ttyACM1′

#### 6.3.3 Homing

The command ‘home()’ will start with the homing process for the defined axis. If required, axis can be added or removed to fit the specific requirements.

def home_all():

robot2._driver.send_command('G28.2 Y Z')

robot2._driver.send_command('G28.2 A')

robot2._driver.send_command('G28.2B')

robot2._driver.send_command('G28.2 X')

robot2._driver.send_command('G90′)

#### 6.3.4 Move to programmed positions

Basic commands are written in the G-code language^24^ to enable customization of movements and make it accessible to anyone with basic programming skills. Examples are provided in the following.

Switch to absolute coordinates:

robot2._driver.send_command('G90′)

Switch to relative coordinates:

robot2._driver.send_command('G91′)

Define movement speed in mm/min

robot2._driver.send_command('G0 F1000′)

Define movement steps

robot2._driver.send_command('G0 X5 Y100 A88′)

robot2._driver.send_command('G0 X0 Y150 A33′)

#### 6.3.5 Advanced control

If specific protocol blocks or general functionalities are repeated from time to time, parameters can be defined at the beginning and called in a function. An example is provided below:

# definitions of module positions for the moduleTransport

transportposition['modulePipetting'] = 'G0 X481 Y5 Z5 A4 B5 F2000′

transportposition['moduleCrosslinker'] = 'G0 X170 Y5 Z5 A4 B5 F2000′

transportposition['moduleStorage'] = 'G0 X21.3 Y5 Z5 A4 B5 F2000′

# definition of crosslinker parameters for the moduleCrosslinker

# used to initiate photo-induced polymerization of hydrogels

lightON = 'M106′

lightOFF = 'M107′

# define function

def crosslinking1min():

# absolute positioning

robot2._driver.send_command('G90′)

# move plate to crosslinker module

robot2._driver.send_command(transportposition['moduleCrosslinker'])

# specifiy intensity

robot2._driver.send_command(intensity_P1)

# LEDs on

robot2._driver.send_command(lightON)

# duration

sleep(60)

# LEDs off

robot2._driver.send_command(lightOFF)

# move plate to pipetting module

robot2._driver.send_command(transportposition['modulePipetting'])

The function can then be called in the main protocol script with:

crosslinking1min()

### 6.4 Operating the workstation

| Plug modules into workstation frame
| Connect peripherals (e.g. stepper motors, end stops) of each module with the control board(s)
| Start the RaspberryPi
| Connect control boards to the computational module via USB (Ethernet is also available, but has not been tested)
| Identify serial port name (e.g. /dev/ttyACM0) via terminal^25^
| Define serial port name in ‘commands.py’
| Write custom protocol script
| Run ‘protocol.py’ script

## 7 Validation and characterization

The following sections describe validation methods to evaluate the performance parameters and present applications in a laboratory setting for end users.

### 7.1 Evaluation of the temperature dock

The performance of the temperature dock was evaluated by (i) assessing the minimum and maximum temperature reachable, and (ii) demonstrating the capability to heat reagents and maintaining a constant sample temperature of 37 °C. This temperature is adjusted for cell culture experiments, as mammalian cells exhibit optimal growth at 37 °C. Temperature changes were recorded with an Arduino Uno connected to waterproof temperature probes (Supplementary Material), which were inserted in 5 mL reaction tubes filled with 3.5 mL water. Temperature changes were recorded for the aluminium block and the sample temperature.

**Fig. 5:**
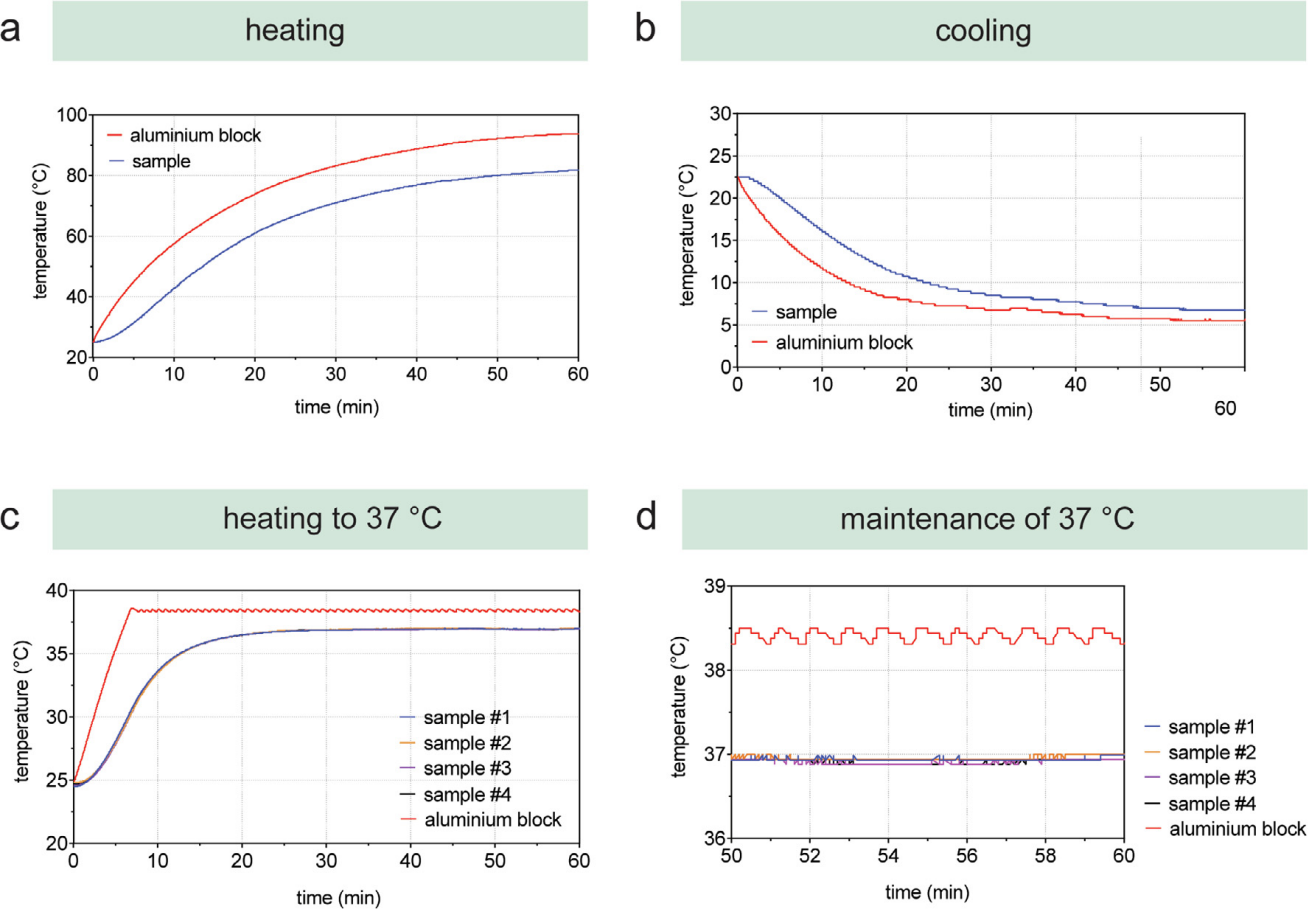
Evaluation of the temperature dock by assessing (a) maximum heating and (b) minimum cooling temperature and the ability to (c) heat to 37 °C and maintain (d) this temperature. All graphs show the temperature of the aluminium block (red) and (a) and (b) indicate the sample temperature (blue). For (c) and (d), the temperature of four samples was recorded and plotted in the graphs.

Minimum and maximum temperature measurement showed a temperature of 5.5 °C and 95 °C, respectively (Fig. 5/a,b). When heating to 37 °C, temperature changes exhibited changes of 0.1 °C between the four samples and the experiment demonstrated the ability to maintain 37 °C over time (Fig. 5/c,d). The experiment was started at room temperature; hence the steel blocks were not pre-heated at 37°when the tubes with water were inserted and the experiment started. Pre-heated steel blocks would result in a significant reduction of the heating time. The integration of temperature docks with a temperature range of 5.5 °C and 95 °C increase the application range for material research, such as the processing of thermoresponsive hydrogels. For example, matrigel and collagen solutions are cooled while GelMA is heated to allow pipetting and the processing of these thermoresponsive materials.

### 7.2 Evaluation of the motorized positioning system

The suitability of any motorized positioning system for a given application is determined by its accuracy and repeatability. To evaluate whether the targeted position equals the actual position of the positioning system, the accuracy and repeatability of the transport and pipetting module are assessed by repeatedly moving one axis with set step size and subsequently acquiring an image of a test target with a defined line pattern [30].

The accuracy and repeatability of the positioning system were assessed according to ISO 230-2:2014^26^ which specifies procedures for testing and evaluating the accuracy and repeatability of the positioning of numerically controlled axes. In accordance with ISO 230, positioning repeatability is the range of the positional deviations obtained by a series of unidirectional movement to a target position. The positioning deviation is the position reached by the functional point on the moving component minus the target position. The accuracy describes the difference between the targeted position as specified in the protocol script and the actual position as quantified by a measurement device.

Positioning accuracy, also referred to as the systematic error, is calculated by:

e_s_ = $\overset{¯}{x}$ − $x_{\mathrm{test}}$

e_s_ systematic error (in μm)

$\overset{¯}{x}$arithmetic mean of measured positional deviations (in μm)

$x_{\mathrm{test}}$targeted position (in μm)

Positioning repeatability, also referred to as random error, is calculated by:

e_r_ = s = $\sqrt{\frac{\sum_{i=1}^{n}{\left(x_{i}-\overset{-}{x}\right)}^{2}}{n-1}}$

e_r_ random error (in μm)

s sample standard deviation

$\overset{¯}{x}$arithmetic mean of measured positional deviations (in μm)

$x_{i}$individual measured positional deviations (in μm)

n number of measurements

To evaluate the accuracy and repeatability of the transport module, the axis was operated in unidirectional mode by repeatedly moving 40 times from the zero-point calibration to the three module positions located at x = 7 cm (storage module), x = 170 cm (photo-crosslinker module), and x = 480 cm (pipetting module). At each module position, an image of a USAF 1951 test target was acquired with a Raspberry Pi Camera Board (V2, Raspberry Pi Foundation, Cambridge, UK) connected to a Raspberry Pi (Raspberry Pi 3 Model B + ). Exported tiff images are then uploaded and processed with ImageJ (National Institutes of Health) using the plugin Register Virtual Stack Slices [59]. Here, images were imported, and the displacement information (in pixels) was calculated to a pre-defined reference image, which was the first image taken for the respective analysis run. Unidirectional registration information was exported by the plug-in to *name*.xlm-files (XML: Extensible Markup Language), and pixel information was converted into micrometres using a conversion factor, which was determined by imaging patterns with defined dimensions of the USAF 1951 test target. The positioning system of the pipetting module was evaluated accordingly by moving the x-, y-, and z-axis 25 times to a predefined well position on the deck. Python scripts for the measurement protocols can be found in the Zenodo repository. Table 3 summarizes the mean and standard deviation for both experiments.

**Table 3:** Summary of the measurement data for the evaluation of the accuracy and repeatability of the positioning system.

Unit	Targeted position	Mean	Standard deviation
Transport module			
	Storage module	0.8 µm	± 5.1 µm
	Crosslinker module	23.4 µm	± 33.9 µm
	Pipetting module	51.2 µm	± 102.3 µm

Pipetting module			
	X-direction	67.0 µm	± 37.8 µm
	Y-direction	−97.0 µm	± 50.2 µm
	Z-direction	−47.8 µm	± 34.8 µm

**Fig. 6:**
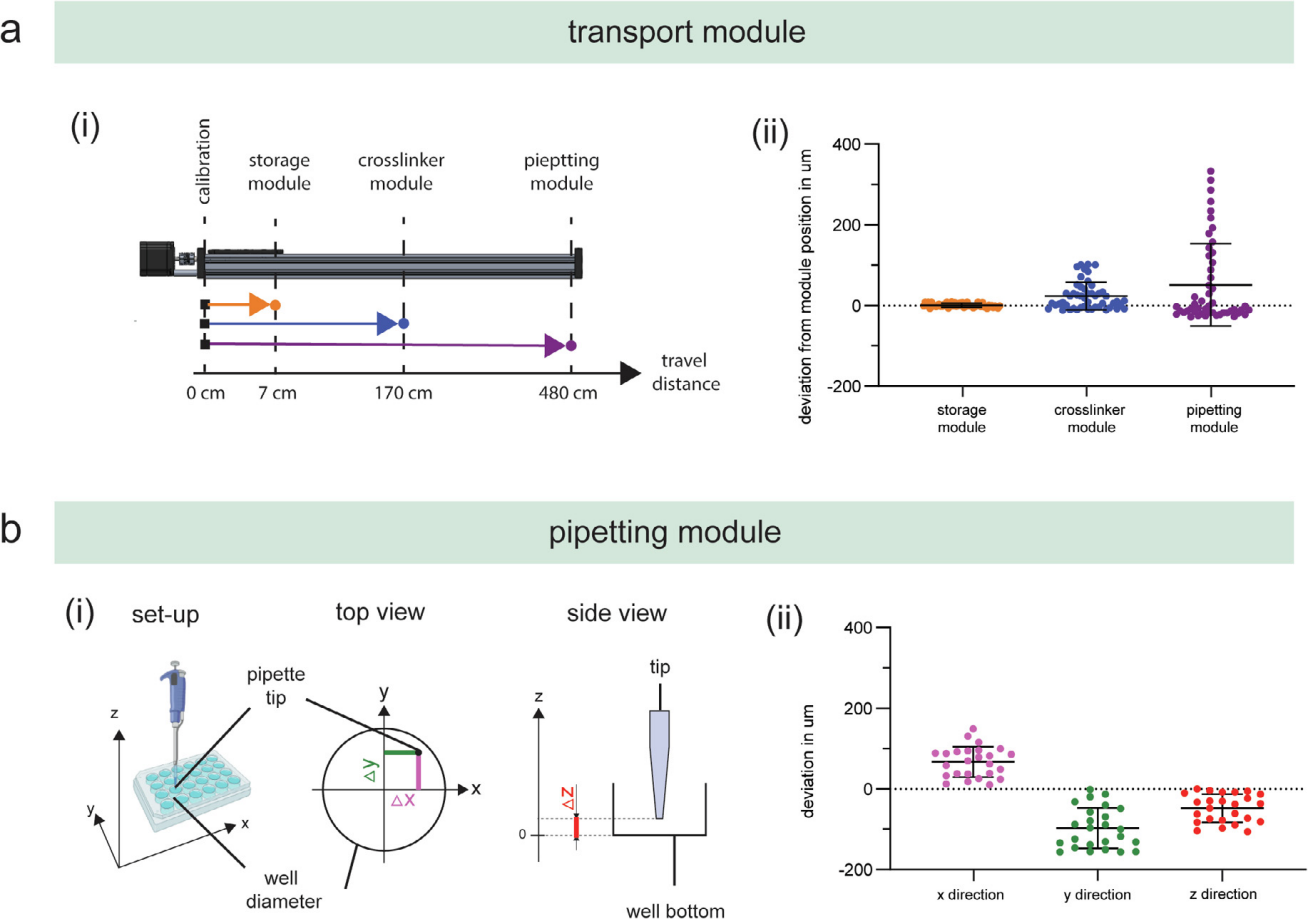
Measurements of the accuracy and repeatability for (a) the transport module and (b) the pipetting module.

Distribution of the measured deviation from the set module positions is displayed with the standard deviation and the single data points (Fig. 6/a). The transportation module exhibited average deviations of 0.8 µm (±5.1 µm) for the storage module, 23.4 µm (±33.9 µm) for the crosslinker module, and 51.2 µm (±102.3 µm) for the pipetting module. Increased standard deviation from the storage to the pipetting module can be explained with the increasing travel range from 7 to 480 cm. Manufacturers of commercial linear stages have acknowledged previously that greater travel distance will generally have poorer accuracy over their full range.^27^ However, there is no standard which defines acceptable stage accuracy related to travel distance, and, therefore, the acceptable stage accuracy is mostly defined by the final application. For the presented case study, the measured stage accuracy is acceptable to guarantee accurate transportation of cell culture vessels.

The average deviation from the set pipette tip position to the associated measured position was calculated for x-, y-, and z-direction (Fig. 6/b). The minimal and maximal deviation from the set pipette tip position alters between 10.2 and 148.8 µm for the x-direction, −156.3 and −1.4 µm for the y-direction, and −105.9 and 0.0 µm for the z-direction. The pipetting module exhibited an accuracy and standard deviation of 67.0 µm (±37.8 µm) for x-direction, −97.0 µm (±50.2 µm) for y-direction, and −47.8 µm (±34.8 µm) for z-direction. These results are well within the defined limits of ± 300 µm for accuracy and ± 100 µm for repeatability as used in comparable commercial solutions, such as the Eppendorf epMotion® 5070.^28^ In conclusion, the evaluation of the motorized positioning system demonstrates sufficient accuracy and repeatability for pipetting and sample transport functionality. If high resolution is required for a specific application (e.g. imaging solutions), greater accuracy and repeatability can be achieved by the implementation of open source motorized positioning system for microscopic applications [30] or commercial stages.

### 7.3 Automated preparation of concentration gradients

**Fig. 7:**
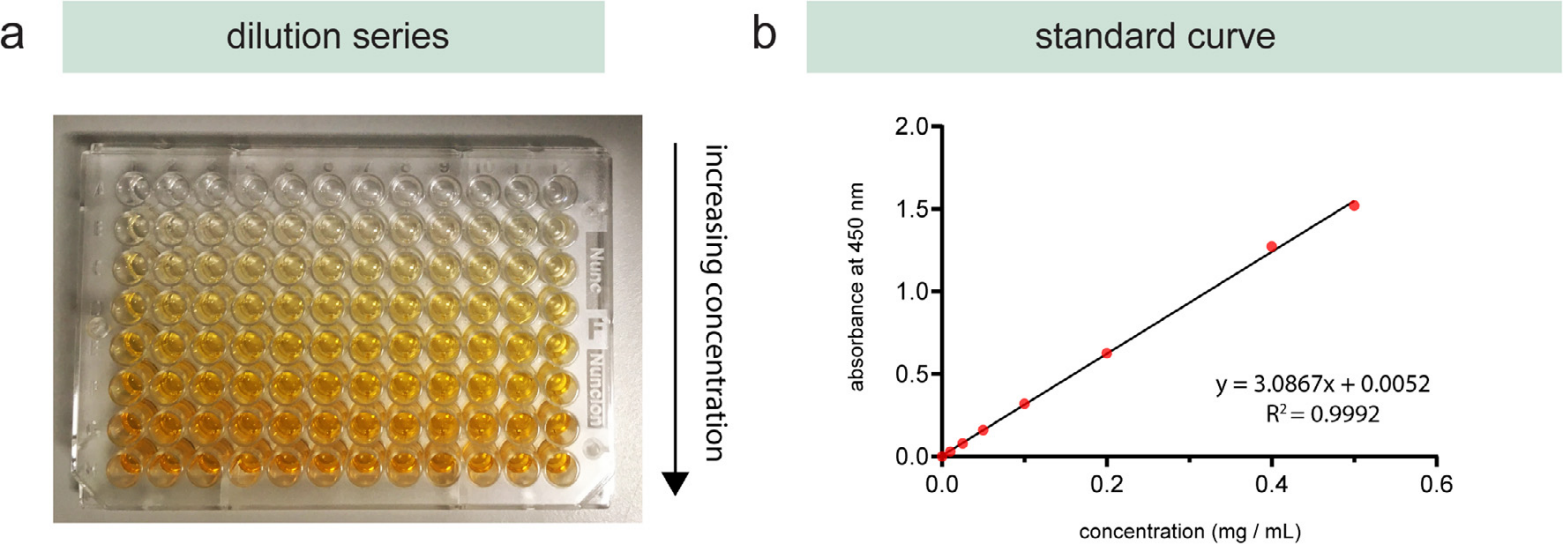
Automated generation of (a) dilution series of OrangeG and (b) the calculated standard curve with an R^2^ value of 0.9992.

The preparation of concentration gradients for reagents (e.g. drugs, standard curves) is another repetitive and simple task in life science laboratories. While concentration gradients are essential for various wet-lab experiments, they are very time-consuming and manual steps increase the probability of human error. We demonstrated the suitability of the presented workstation for automated preparation of concentration gradients by preparing sequential dilution of a substance and transferring them into a 96-well plate. A seven-step serial dilution series of OrangeG was prepared in a flat-bottom 96-well microtiter plate and absorbance measurements were conducted with a spectrophotometer at 450 nm [60]. The standard curve was prepared in Excel by averaging the absorbance readings from the standards and calculating the curve using linear regression (Fig. 7). The coefficient of determination, also referred to as R^2^ value, of 0.9992 showed that the acquired data fit the regression line very closely and illustrates the high reliability of the serial dilution. This serial dilution experiment demonstrates the suitability to automate the preparation for such an experimental setup to provide scientists with reliable results and time-efficient workflows. An extensive data set on the reproducibility of preparing hydrogel dilution series is provided in Eggert et al. [61].

### 7.4 Automated media change for 2D cell culture

**Fig. 8:**
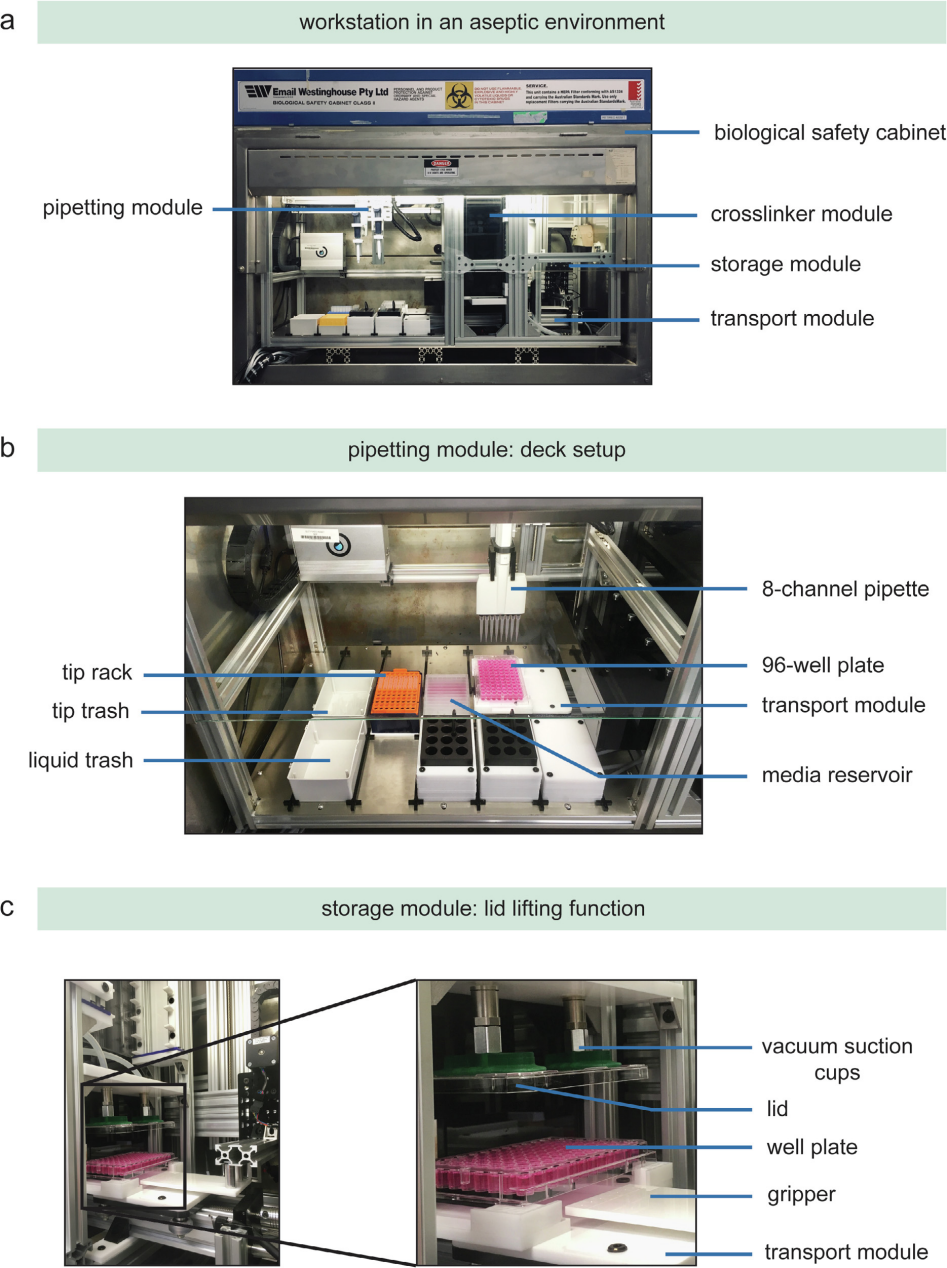
(a) Deck setup of the pipetting module and (b) the lid lifting function. (i) The gripper unit gets the plate from the rack unit, (ii, iii) positions the plate under the vacuum suction cups and (iv) placing the plate without the lid on the transport module.

To demonstrate the automation potential of the setup for 2D cell culture processes, we scripted an experiment to carry out media change. This task, in particular, involves rather simple and repetitive execution steps (e.g. pipetting) and does not require human intervention *per se*. Breast cancer cells (MDA-MB-231) were cultured according to ATCC (American Type Culture Collection) guidelines and subsequently seeded as 2D monolayers with a cell density of 5000 cells per well in 96-well plates. The deck setup of the pipetting module is shown in Fig. 8. The scripted protocol successfully lifted the lid of the well plate, transported the well plate from the storage module to the pipetting module, and performed media change day by removing and adding 50% of the total media volume of 200 μl (Supplementary Video 3). Cell imaging on day 1 and day 6 showed no contamination issues and demonstrated spreading of cells on the well bottom.

### 7.5 Automated manufacturing of hydrogel constructs and preparation of hydrogel concentration gradients

**Fig. 9:**
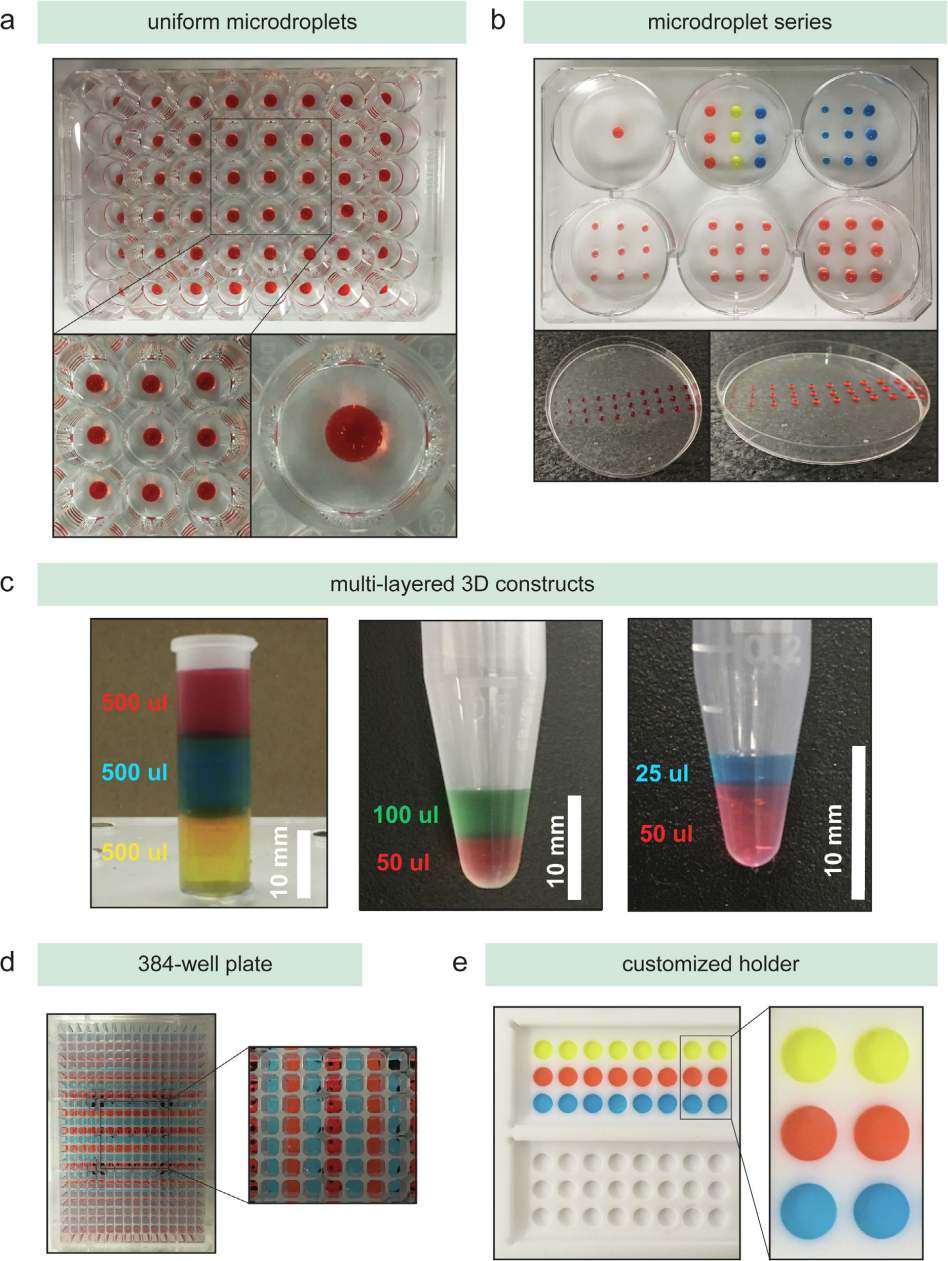
Illustration of the manufacturing capabilities with 10% (w/v) GelMA, stained with food colours: (a) uniform microdroplets, (b) microdroplet series, (c) multi-layered 3D constructs, (d) processing of a 384-well plate format, and (e) customized holders such as the Teflon plate.

The pipetting of hydrogels is challenging due to the viscosity of the materials and changes in their physical properties with temperature. To demonstrate the functionality of the developed setup, the temperature docks were integrated for heating 10% (w/v) Gelatin methacryloyl (GelMA) hydrogel precursor solutions to 37 °C to, subsequently, manufacture hydrogel-based 3D constructs. As a simple example, a GelMA microdroplet series was manufactured ranging from 1 to 10 μl into a Petri dish as well as a 4 × 4 microdroplet array into 6-well plate (Fig. 9/a, b). Building upon the functionality to dispense droplets, multi-layered 3D constructs were manufacturing with various volumes (Fig. 9/c). Finally, the ability to process a 384-well plate and customized vessel holders were demonstrated with 10% GelMA as a candidate hydrogel (Fig. 9/d, e). Since most commercial liquid handling robots are not able to process viscous materials in a reliable manner, dispensing and aspirating tasks of such materials is still mostly done manually. In contrast to liquid handling robots with air-displacement pipettes, the integration of positive-displacement pipettes enables reliable pipetting of viscous materials in various modes. Additional information and data on the operation of the workstation, the usage of an open-source protocol designer, and the validation and verification to identify reproducible hydrogel mixtures is available in Eggert et al. [61].

### 7.6 Monitoring of roundness factor and surface area of hydrogel-based microdroplets

**Fig. 10:**
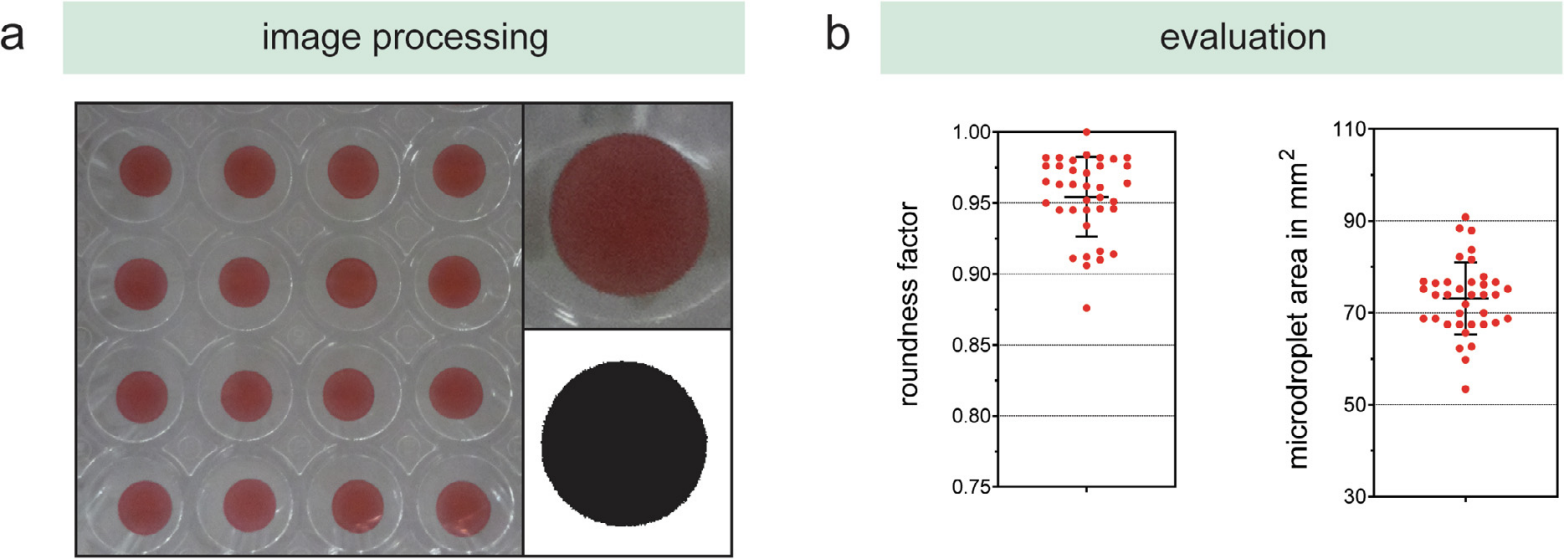
Microdroplet monitoring capabilities with the subsequent (a) image processing workflow and the (b) calculated roundness factor and microdroplet area.

To evaluate the reliability of the microdroplet manufacturing process, a camera-based solution was implemented to monitor the roundness factor and the 2D surface area of the hydrogel-based microdroplets. Within a proof-of-concept study, 10% (w/v) GelMA-based 25 μL microdroplets were manufactured and subsequently images using a Raspberry Pi Camera Board, which was implemented in the storage module. After converting single droplets to a binary image, roundness factor and surface area were calculates using a custom-written image analysis script in ImageJ (Fig. 10). Calculate roundness and area factors yielded 0.954 ± 0.028 and 97.145 ± 7.754, respectively. Especially the roundness factor demonstrates successfully the capability to manufacture reproducible microdroplets in a defined range. Although the roundness factor and the 2D surface area are an indicator for the microdroplet reproducibility, the two factors do not indicate changes in the volume.

## 8 Conclusion and future work

Automation in Life Science research enables precise manipulation and investigation of samples to generate reliable data in a time-efficient manner. However, current workflows for wet-lab experiments are mostly executed manually in a low-throughput manner, resulting in time-consuming experiments and a high probability of human error. To address these challenges, we presented the OpenWorkstation concept, which is inspired by modular assembly lines, to enable the development of a platform with customized hardware modules specifically designed to experimental requirements. This set-up allows a scalable, easily reconfigurable, and cost-effective solution to design and develop scientific instrumentation for academic groups, start-ups, makerspaces, and FabLabs. This report includes a description of the concept, including a Systems Engineering framework to approach the hardware development, and presents the successful adoption for automated 2D and 3D cell culture workflows in a case study (Table 4). In the future, we hope that the presented open source concept will be applied to automate experiments for 2D and 3D cell culture operations as well as be used as an educational tool for open source workshops [62], [63]. We hope that the concept will be adopted by a broad community of users to design and customize hardware modules, and freely share design files and experimental code.

**Table 4:** Summary of module specifications and features. ANSI: American National Standards Institute, SLAS: Society for Laboratory Automation and Screening.

Module	Features/specification
Pipetting module	- High positioning accuracy and repeatability: ± 100 µm - Pipetting range from 1 to 1000 µl - Operation of two standard laboratory pipettes (single- or 8-channel pipette) - 8-deck capacity according to ANSI/SLAS standards - Compatible with tubes (0.2 mL to 50 mL), microplates with up to 384 wells and reservoirs (up to 200 mL)

Photo-crosslinker module	- Exchangeable LED panel with different wavelengths - Adjustable crosslinking intensity - Integrated sliding mask to create exposure gradients

Storage module	- Removable rack unit - Storage capacity with up to six well plates - Customizable rack design

Transport module	- High positioning accuracy and repeatability: ± 100 µm - Compatible with microplates designed according to ANSI/SLAS standards

## 9 Human and animal rights

The work did not involve the use of any human or animal subjects. Cell culture work with MDA cells has been carried out in accordance with the required guidelines.

## Declaration of Competing Interest

The authors declare that they have no known competing financial interests or personal relationships that could have appeared to influence the work reported in this paper.

## Footnotes

2 https://www.duet3d.com

3 https://www.panucatt.com/azteeg_X5_GT_reprap_3d_printer_controller_p/ax5gt.htm

4 https://core-electronics.com.au/tinyg-cnc-controller-board-v8.html

5 https://www.youtube.com/watch?v=tLozVt_CjXQ

6 https://www.youtube.com/watch?v=OKe5Pa23JrI

7 https://www.youtube.com/watch?v=t7mnlmPzgSQ

8 https://learn.sparkfun.com/tutorials/electric-power/all

9 https://learn.adafruit.com/adafruit-guide-excellent-soldering

10 https://learn.sparkfun.com/tutorials/working-with-wire/all

11 https://learn.adafruit.com/wires-and-connections

18 https://docs.python.org/3/tutorial/venv.html

20 http://smoothieware.org/configuration-options

21 https://thonny.org

22 https://docs.python.org/3/library/idle.html

23 https://docs.opentrons.com/ot1/index.html

24 https://reprap.org/wiki/G-code

26 https://www.iso.org/standard/55295.html

27 https://www.zaber.com/technical-articles/actuator-precision-characterization

28 https://online-shop.eppendorf.com.au/AU-en/Automated-Pipetting-44509/Liquid-Handling-Workstations-44510/epMotion5070-PF-68886.html

## Footnotes

12 https://www.python.org/downloads/

13 https://projects.raspberrypi.org/en/projects/raspberry-pi-setting-up

14 https://projects.raspberrypi.org/en/projects/raspberry-pi-setting-up/5

15 https://projects.raspberrypi.org/en/projects/raspberry-pi-using/4

16 https://pypi.org/project/pip/

17 https://docs.python.org/3/library/venv.html

19 https://docs.opentrons.com/ot1/index.html

25 https://learn.adafruit.com/welcome-to-circuitpython/advanced-serial-console-on-mac-and-linux

## References

[1] Vasilevsky N.A., Brush M.H., Paddock H., Ponting L., Tripathy S.J., LaRocca G.M., Haendel M.A. (2013). On the reproducibility of science: unique identification of research resources in the biomedical literature. PeerJ.

[2] Jarvis M.F., Williams M. (2016). Irreproducibility in preclinical biomedical research: perceptions, uncertainties, and knowledge gaps. Trends Pharmacol Sci..

[3] Foundation National Science 2015 Reproducibility, Replicability, and Generalization https://www.nsf.gov/sbe/SBE_Spring_2015_AC_Meeting.

[4] Niepel M., Hafner M., Mills C.E., Subramanian K., Williams E.H., Chung M., Gaudio B., Barrette A.M., Stern A.D., Hu B., Korkola J.E., Gray J.W., Birtwistle M.R., Heiser L.M., Sorger P.K., Shamu C.E., Jayaraman G., Azeloglu E.U., Iyengar R., Sobie E.A., Mills G.B., Liby T., Jaffe J.D., Alimova M., Davison D., Lu X., Golub T.R., Subramanian A., Shelley B., Svendsen C.N., Ma’ayan A., Medvedovic M., Feiler H.S., Smith R., Devlin K.A. (2019). A multi-center study on the reproducibility of drug-response assays in mammalian cell lines. Cell Syst*.*.

[5] Mullard A. (2017). Cancer reproducibility project yields first results. Nat. Rev. Drug Discov..

[6] Collins F.S., Tabak L.A. (2014). Policy: NIH plans to enhance reproducibility. Nature.

[7] Frye S.V., Arkin M.R., Arrowsmith C.H., Conn P.J., Glicksman M.A., Hull-Ryde E.A., Slusher B.S. (2015). Tackling reproducibility in academic preclinical drug discovery. Nat. Rev. Drug Discov..

[8] Baker M. (2016). 1,500 scientists lift the lid on reproducibility. Nature.

[9] Ioannidis J.P.A. (2005). Why most published research findings are false. PLoS Med..

[10] Kim B., Trounson A. (2018). Design preclinical studies for reproducibility. Nat Biomed. Eng..

[11] Sadowski M.I., Grant C., Fell T.S. (2016). Harnessing QbD, programming languages, and automation for reproducible biology. Trends Biotechnol..

[12] King R.D., Rowland J., Oliver S.G., Young M., Aubrey W., Byrne E., Liakata M., Markham M., Pir P., Soldatova L.N., Sparkes A., Whelan K.E., Clare A. (2009). The Automation of Science. Science.

[13] Chapman T. (2003). Lab automation and robotics: automation on the move. Nature.

[14] Blow N. (2008). Lab automation: tales along the road to automation. Nat. Methods.

[15] Bédard A., Adamo A., Aroh K.C., Russell M.G., Bedermann A.A., Torosian J., Yue B., Jensen K.F., Jamison T.F. (2018). Reconfigurable system for automated optimization of diverse chemical reactions. Science.

[16] Check Hayden E., Carolina N. (2014). The automated lab. Nature.

[17] Eggert S., Hutmacher D.W. (2019). In vitro disease models 4.0 via automation and high-throughput processing. Biofabrication.

[18] Kohn J., Welsh W.J., Knight D. (2007). A new approach to the rationale discovery of polymeric biomaterials. Biomaterials.

[19] Pearce J.M. (2014). Cut costs with open-source hardware. Nature.

[20] Powell A. (2012). Democratizing production through open source knowledge: from open software to open hardware, media. Cult. Soc..

[21] Pearce J.M. (2012). Building research equipment with free open-source hardware. Science.

[22] Pearce J M 2014 Chapter 1: Introduction to Open-Source Hardware for Science; in Open-Source Lab - How to Build Your Own Hardware and Reduce Research Costs (Elsevier Inc.) pp 1–11.

[23] Zhang C., Wijnen B., Pearce J.M. (2016). Open-source 3-D platform for low-cost scientific instrument ecosystem. J. Lab. Autom..

[24] Attaran M. (2017). The rise of 3-D printing: the advantages of additive manufacturing over traditional manufacturing. Bus Horiz..

[25] Nejatimoharrami F., Faina A., Stoy K. (2017). New capabilities of EvoBot: a modular, open-source liquid-handling robot. SLAS Technol. Transl. Life Sci. Innov..

[26] Barthels F., Barthels U., Schwickert M., Schirmeister T. (2020). FINDUS: an open-source 3D printable liquid-handling workstation for laboratory automation in life sciences. SLAS Technol. Transl. Life Sci. Innov..

[27] Steffens S., Nüßer L., Seiler T.B., Ruchter N., Schumann M., Döring R., Cofalla C., Ostfeld A., Salomons E., Schüttrumpf H., Hollert H., Brinkmann M. (2017). A versatile and low-cost open source pipetting robot for automation of toxicological and ecotoxicological bioassays. PLoS ONE.

[28] Enten A., Yang Y., Ye Z., Chu R., Van T., Rothschild B., Gonzalez F., Sulchek T. (2016). A liquid-handling robot for automated attachment of biomolecules to microbeads. J. Lab. Autom..

[29] Carvalho M.C., Osmar Murray R.H. (2018). the open-source microsyringe autosampler. HardwareX.

[30] Schneidereit D., Kraus L., Meier J.C., Friedrich O., Gilbert D.F. (2017). Step-by-step guide to building an inexpensive 3D printed motorized positioning stage for automated high-content screening microscopy. Biosens. Bioelectron..

[31] Campbell R A A, Eifert R W, Turner G C, Openstage (Eds.), A Low-Cost Motorized Microscope Stage with Sub-Micron Positioning Accuracy, PLoS One 9 (2014) e88977. 10.1371/journal.pone.0088977 PMC3935852 24586468

[32] Nuñez I., Matute T., Herrera R., Keymer J., Marzullo T., Rudge T., Federici F.F. (2017). Low cost and open source multi-fluorescence imaging system for teaching and research in biology and bioengineering. PLoS ONE.

[33] Bohm A. (2019). AMi: a GUI-based, open-source system for imaging samples in multi-well plates. Acta Crystallogr. Sect. F Struct. Biol. Commun..

[34] Li H., Soto-Montoya H., Voisin M., Valenzuela L.F., Prakash M., Octopi (2019). Open configurable high-throughput imaging platform for infectious disease diagnosis in the field. bioRxiv.

[35] Wijnen B., Hunt E.J., Anzalone G.C., Pearce J.M. (2014). Open-source syringe pump library ed G F Gilestro. PLoS ONE.

[36] Kassis T., Perez P.M., Yang C.J. W., Soenksen L.R., Trumper D.L., Griffith L.G. (2018). PiFlow, A biocompatible low-cost programmable dynamic flow pumping system utilizing a Raspberry Pi Zero and commercial piezoelectric pumps. HardwareX.

[37] Almada P., Pereira P.M., Culley S., Caillol G., Boroni-Rueda F., Dix C.L., Charras G., Baum B., Laine R.F., Leterrier C., Henriques R. (2019). Automating multimodal microscopy with NanoJ-Fluidics. Nat. Commun..

[38] Klar V., Pearce J.M., Kärki P., Kuosmanen P. (2019). Ystruder: open source multifunction extruder with sensing and monitoring capabilities. HardwareX.

[39] Kong D.S., Thorsen T.A., Babb J., Wick S.T., Gam J.J., Weiss R., Carr P.A. (2017). Open-source, community-driven microfluidics with Metafluidics. Nat. Biotechnol..

[40] Brower K., Puccinelli R.R., Markin C.J., Shimko T.C., Longwell S.A., Cruz B., Gomez-Sjoberg R., Fordyce P.M. (2018). An open-source, programmable pneumatic setup for operation and automated control of single- and multi-layer microfluidic devices. HardwareX.

[41] Watson C., Senyo S. (2019). All-in-one automated microfluidics control system. HardwareX.

[42] Wong B.G., Mancuso C.P., Kiriakov S., Bashor C.J., Khalil A.S. (2018). Precise, automated control of conditions for high-throughput growth of yeast and bacteria with eVOLVER. Nat. Biotechnol..

[43] Steel H., Habgood R., Papachristodoulou A. (2019). Chi.Bio: An open-source automated experimental platform for biological science research. bioRxiv.

[44] Malda J., Visser J., Melchels F.P., Jüngst T., Hennink W.E., Dhert W.J.A., Groll J., Hutmacher D.W. (2013). 25th anniversary article: engineering hydrogels for biofabrication. Adv Mater..

[45] Annabi N., Tamayol A., Uquillas J.A., Akbari M., Bertassoni L.E., Cha C., Camci-Unal G., Dokmeci M.R., Peppas N.A., Khademhosseini A. (2014). 25th anniversary article: rational design and applications of hydrogels in regenerative medicine. Adv Mater..

[46] Kratochvil M.J., Seymour A.J., Li T.L., Paşca S.P., Kuo C.J., Heilshorn S.C. (2019). Engineered materials for organoid systems. Nat. Rev. Mater..

[47] Horvath P., Aulner N., Bickle M., Davies A.M., Del N.E., Ebner D., Montoya M.C., Östling P., Pietiäinen V., Price L.S., Shorte S.L., Turcatti G., von Schantz C., Carragher N.O. (2016). Screening out irrelevant cell-based models of disease. Nat. Rev. Drug Discov..

[48] US Department of Defense Systems Management College 2001 Systems engineering fundamentals ed Defense Acquisition University Press (Fort Belvoir, Virginia, USA, Virginia, USA: Systems Management College).

[49] Ottino J.M. (2004). Engineering complex systems. Nature.

[50] Keating C.B., Padilla J.J., Adams K. (2008). System of systems engineering requirements: challenges and guidelines. Eng Manage. J..

[51] Wasson C.S. (2005).

[52] Ulrich K.T., Eppinger S.D. (2012).

[53] Sosale S., Hashemian M., Gu P. (1997). Product modularization for reuse and recycling. Concurrent Product Design and Environmentally Conscious Manufacturing: Presented at the 1997 Asme International Mechanical Engineering Congress and Exposition.

[54] Pearce J.M. (2016). Return on investment for open source scientific hardware development. Sci Public Policy.

[55] May M. (2019). A DIY approach to automating your lab. Nature.

[56] Virtanen P., Gommers R., Oliphant T.E., Haberland M., Reddy T., Cournapeau D., Burovski E., Peterson P., Weckesser W., Bright J., van der Walt S.J., Brett M., Wilson J., Millman K.J., Mayorov N., Nelson A.R.J., Jones E., Kern R., Larson E., Carey C.J., Polat İ., Feng Y., Moore E.W., VanderPlas J., Laxalde D., Perktold J., Cimrman R., Henriksen I., Quintero E.A., Harris C.R., Archibald A.M., Ribeiro A.H., Pedregosa F., van Mulbregt P. (2020). SciPy 1.0: fundamental algorithms for scientific computing in Python. Nat. Methods.

[57] Woelfle M., Olliaro P., Todd M.H. (2011). Open science is a research accelerator. Nat. Chem..

[58] Iglehart B. (2018). MVO automation platform: addressing unmet needs in clinical laboratories with microcontrollers, 3D printing, and open-source hardware/software. SLAS Technol. Transl. Life Sci. Innov..

[59] Arganda-Carreras I., Sorzano C.O.S., Marabini R., Carazo J.M., Ortiz-de-Solorzano C., Kybic J. (2006). International Workshop on Computer Vision Approaches to Medical Image Analysis.

[60] Stangegaard M., Hansen A.J., Frøslev T.G., Morling N. (2011). A simple method for validation and verification of pipettes mounted on automated liquid handlers. J. Lab. Autom..

[61] Eggert S., Kahl M., Kent R., Bock N., Meinert C., Hutmacher D.W. (2020). An Open Source Technology Platform to Manufacture Hydrogel-Based 3D Culture Models in an Automated and Standardized Fashion. J. Vis. Exp..

[62] Gerber L.C., Calasanz-Kaiser A., Hyman L., Voitiuk K., Patil U., Riedel-Kruse I.H. (2017). Liquid-handling Lego robots and experiments for STEM education and research. PLoS Biol*.*.

[63] Huang A., Nguyen P.Q., Stark J.C., Takahashi M.K., Donghia N, Ferrante T, Dy A.J., Hsu K.J., Dubner R.S., Pardee K, Jewett M.C., Collins J.J. (2018). BioBitsTM Explorer: A modular synthetic biology education kit. Sci Adv.

