# Supplementary material for: OpenWorkstation: A modular open-source technology for automated in vitro workflows
Source: HardwareX. 2020 Oct 20;8:e00152. doi: 10.1016/j.ohx.2020.e00152 (PMC9041211; doi:10.1016/j.ohx.2020.e00152)
Supplement: Supplementary data 1 [file mmc1.pdf]

Step-by-Step Build Setup Guide

Table of Contents

1 Base 2

2 Transportation module 2

3 Crosslinker module 4

4 Storage module 6

5 Electrical control box 8

6 List of tools 11

1 Base

The assembly process for the base is depicted in Figure 1.

Depending on the support (e.g. table, lab bench, biological safety cabinet, etc), select an appropriate solution to connect the workstation with the support. If the workstation is to be placed in a biological safety hood, keep the available height of the internal dimensions in mind.

Timing 1 – 2 hours

Materials 30x30mm aluminium profiles, screws (M5, M6), clamping angles, tee nuts for M5 and M6, laser cut parts (specifications are listed in the BOM)

Equipment M5, M6 Allen key

Critical cutting surface of aluminium profile should be straight

1| Laser cut acrylic plates

2| Position two aluminium profiles (30x30mm, 1540mm) next two each other

3| Connect a grid base structure with four aluminium profiles (30x30mm, 400mm) to the base with 29x29 clamping angles using M6x15 screws and M6 nuts

3| Attached spacer plates to the grid base structure using M5x15 screws and M5 nuts.

2 Transportation module

The assembly process for the transport module is depicted in Figure 1.

This module transports sample to the installed modules using a linear actuator and a sample holder. Depending on the overall length of the workstation, the length of the transportation module has to be selected to ensure that the sample can be transported to each module. The sample holder design can be optimized to the required experimental applications.

Timing 1 day

Materials linear actuators, M5 screws, corner brackets, tee nuts for M5, laser cut parts (specifications are listed in the BOM)

Equipment M5 Allen key

Critical cutting surface of aluminium profile should be straight

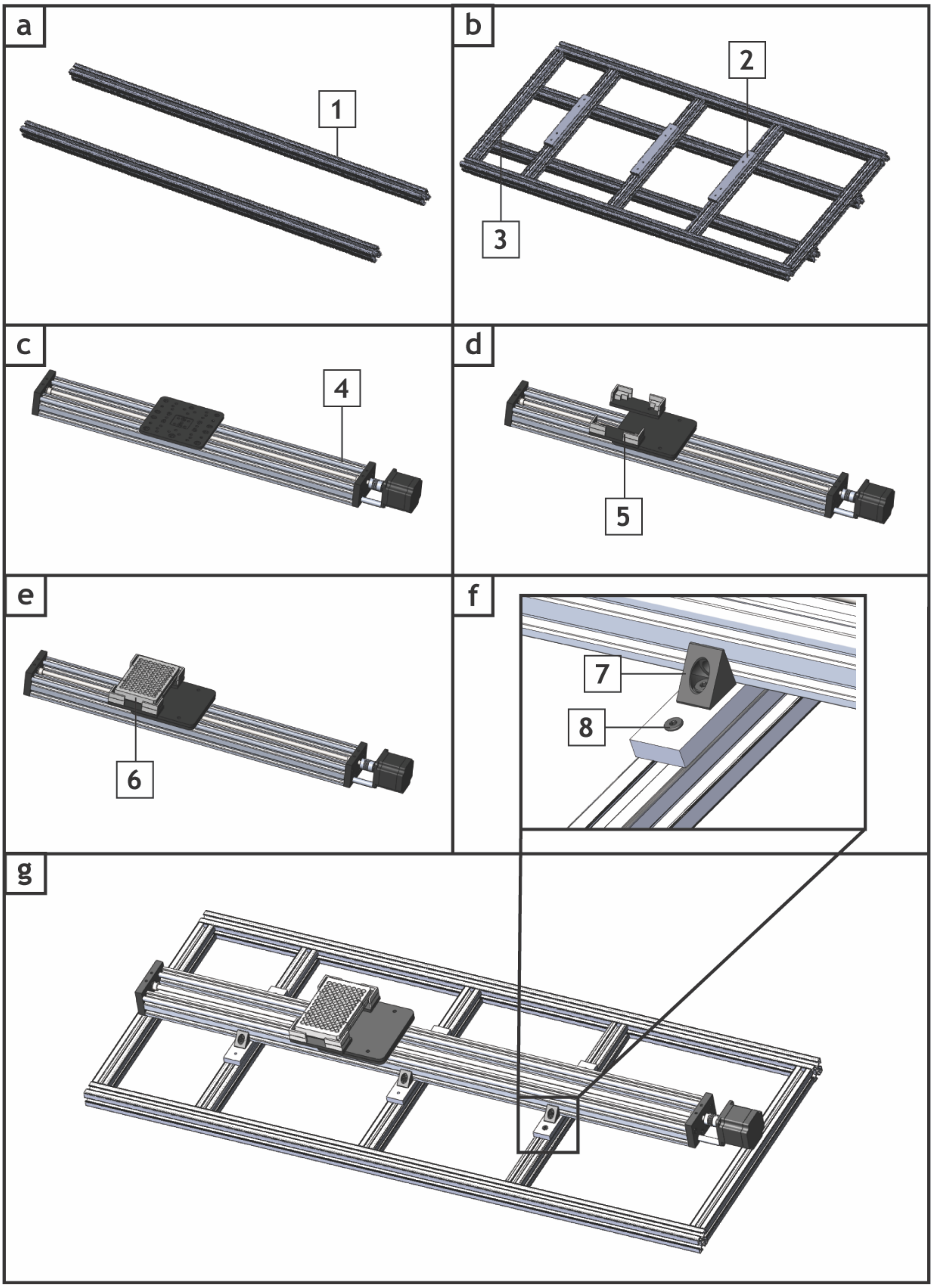

Figure 1: Assembly process for base (a,b), transport module (c-e), and connecting both (g). Parts: Two 30x30mm aluminium profiles (1), connecting struts (3) with spacer (2), linear stage (4) with holder (5) for well plate (6), and corner brackets (7) and screws (8).

1| Laser cut acrylic plates as listed in the BOM

2| Assemble the linear actuator (C-Beam Gantry Actuator) as instructed by OpenBuilds[1]

3| Assemble sample holder and attach to gantry plate using M5x15 screws

4| Base und transportation module are connected using corner brackets.

5| Y-direction of transportation module can be adjusted by positioning the plate spacers

3 Crosslinker module

The assembly process for the crosslinker module is depicted in Figure 2.

The crosslinker module provide photo-induced crosslinking and the ability to generate time gradients using a sliding mask. The LED panel provides a plug-and-play mode to enable fast modification and a customized solution.

Timing 3 days

Materials linear actuators for module as specified in BOM, 20x20mm and 30x30mm aluminium profiles, screws (M3, M5, M6), clamping angles, tee nuts for M5 and M6, NEMA23 stepper motors, cable housing, laser cut parts

Equipment M3, M5, M6 Allen key

Critical cutting surface of aluminium profile should be straight

1| Laser cut acrylic plates

2| Assemble the two linear actuators as instructed by OpenBuilds[2]

3| For the sliding mask, connect the sliding plates to the assembled 250mm linear actuator using M5 screws

4| Attach enclosure to assembly using M5x15 screws and M5 tee nuts

[1] https://www.youtube.com/watch?v=tLozVt_CjXQ

[2] https://www.youtube.com/watch?v=tLozVt_CjXQ

5| The module assembly starts by building the base frame using 30x30 mm profiles which are connected with 29x29 clamping angles using M6x15 screws and M6 nuts

6| Attach right side wall to the base frame using M5x15 screw and M5 tee nuts

7| Attach linear actuator to the back of the module, followed by putting in the back and front enclosure plate as well as the LED panel. The LED panel is attached to the gantry plate via two corner brackets

8| Attach end stop holder at the top. Height and position can be customized/optimized later

9| Connect left enclosure plate and missing profile

10| Finish crosslinker assembly by attaching the sliding mask on the left side

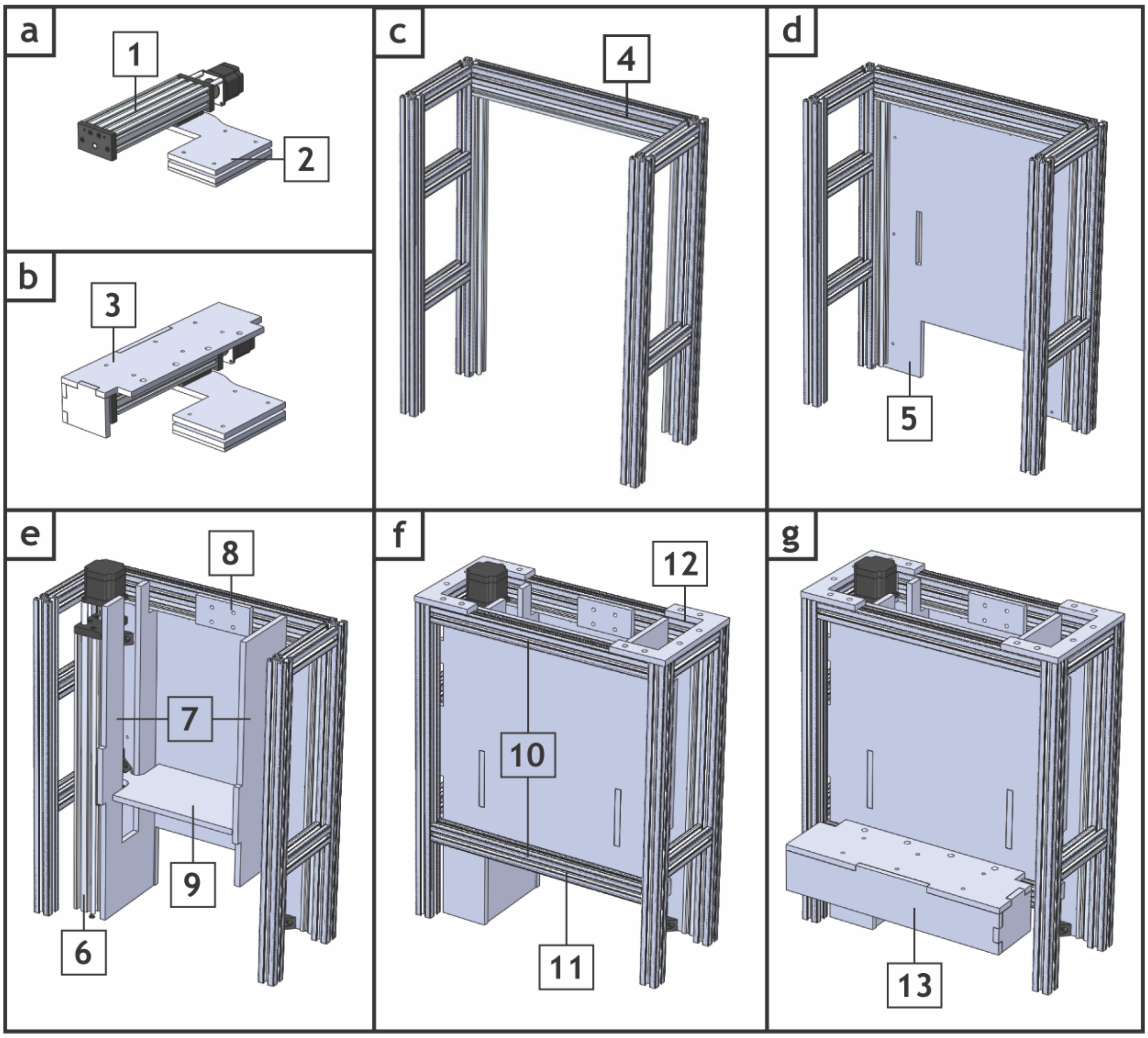

Figure 2: Assembly process for crosslinker module (a-g). Parts: linear stage (1) with mask (2) and enclosure (3), frame (4), side wall (5), linear stage (6), back wall (7), holder for end stop (8), LED panel (9), frame (10), side wall (11), connector plate (12), and gradient slider assembly (13).

4 Storage module

The assembly process for the storage module is depicted in Figure 3.

A storage module has been designed and developed to store well plates or to trash used well plates (e.g. after washing steps). Plates are stored within a rack unit and are positioned onto the transport module by a gripper unit.

Timing 3 – 5 days

Materials linear actuators for module as specified in BOM, 20x20mm and 30x30mm aluminium profiles, screws (M3, M5, M6), clamping angles, tee nuts for M5 and M6, NEMA23 stepper motors, cable housing, laser cut parts

Equipment M3, M5, M6 Allen key

Critical cutting surface of aluminium profile should be straight

1| Laser cut acrylic plates as listed in the BOM

2| Assemble the two linear actuators[3]

3| Assembly gripper unit by connecting the 250mm linear belt & pinion actuator with the 20x20mm profile and the gripper plate. The length of the profile can be adjusted according to the depth of the storage module

4| Assembly rack unit by attaching the four 20x20mm profiles to the laser cut plates which serve as holders for the well plate, and attach side plates (back, left, right)

5| For the main storage module, connect 30x30mm aluminium profiles and insert holding frame for rack unit

6| Connect 500mm linear actuator to the back of the module and attach gripper unit

7| Attach missing profiles on the right side to the base frame

[3] https://www.youtube.com/watch?v=tLozVt_CjXQ

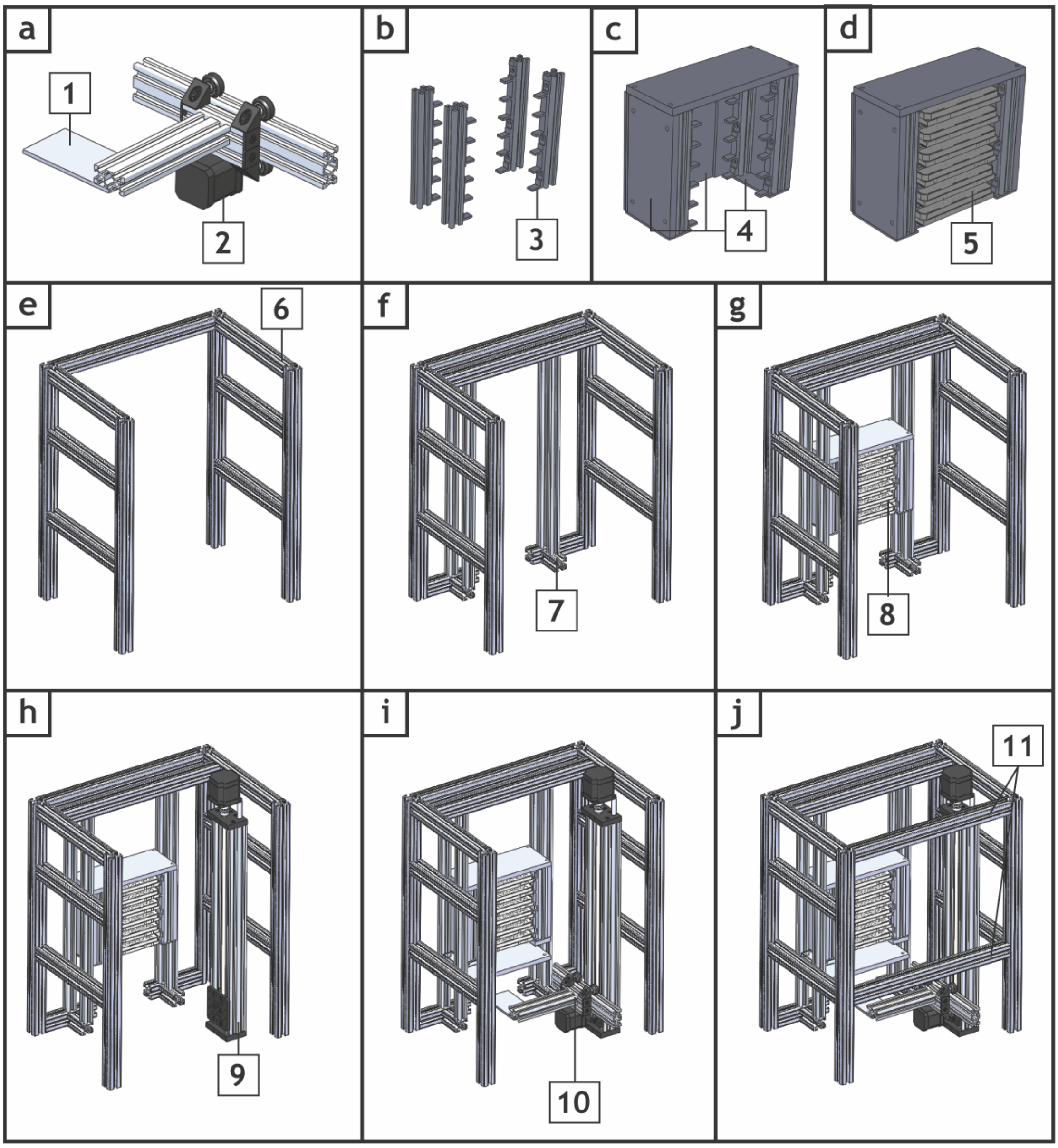

Figure 3: Assembly process for storage module (a-j). Parts: gripper plate (1), linear stage (2), shelf plates (3) for rack unit (4) to store well plates (5), main frame (6), profile frame for rack unit (7), rack unit (8), linear stage (9) with gripper unit (10), and enclosed frame (11).

5 Electrical control box

The main objective of this section is to provide a summary of the electrical work required to connect the stepper motors and end stops as well as to wire the smoothieboard and Arduino (Figure 4). For beginners, introductions to electricity[4], soldering[5] and wiring[6,7] are recommended before starting with the electrical assembly work. A dedicated control box was designed to comply with the requirements of the university's Health and Safety guidelines. The main power circuit is a 12V circuit, which is considered as a low-voltage application in most countries. The number of the required components (e.g. control board) are selected based on the number of hardware modules and the required functionalities (e.g. valve).

Timing 1 week for control box including electrical work

Materials 2 and 4 core cable, solder, solder wick, heat shrink, ferrules crimper, laser cut plates

Equipment fine (or flat tip) soldering iron, tweezers, optical microscope to inspect soldered connections, wire cutters, wire strippers, heat gun, multimeter

Caution use approved or provided ferrules, since ferrule failure can result in wire creeping causing loose wires which may create short circuits

Critical before starting with connecting the control board(s), familiarize yourself with the wiring diagram (see supplements); check with the Health and Safety department, if electrical work can be executed without a license or if qualified industrial electricians are required to connect the components

1| If additional electrical components have been added into the control box, modify the control box geometry to accommodate all parts

2| Laser cut acrylic plates for housing

3| Drill threaded M3 or M5 holes into the base to attach electrical parts

---

[4] https://learn.sparkfun.com/tutorials/electric-power/all

[5] https://learn.adafruit.com/adafruit-guide-excellent-soldering

[6] https://learn.sparkfun.com/tutorials/working-with-wire/all

[7] https://learn.adafruit.com/wires-and-connections

4| Connect electrical parts to the base using M3 and M5 screws, starting with the power supply, control board, Arduino, valve, and breadboards

5| Start to connect electrical parts to each other according to the electrical circuit:

- Check V_{in} and GND connections to prevent a short circuit
- Use provided ferrules or connectors to connect wires

6| Connect main power to power supply, switch power on, and check if any LEDs of the smoothieboard are ON. If not, check troubleshooting list for power supply issues[8]

7| Connect electrical parts of hardware modules to control board

7.1| Connect the stepper motors to the control board.

- Locate A, A', B and B' wires from each of the stepper motors
- Insert wires to the stepper motor top connector according to the provided instructions[9]
- Continue until all stepper motors are connected

7.2| Connect the end stops to the control board

- Locate GND and Signal of each end stop
- Insert wires to the end stop top connector according to the provided instruction[10]
- Continue until all end stops are connected

7.3| Connect valve and LEDs to the control board

- Locate GND and V_{in} of each part
- Insert wires to MOSFET connectors

8| Connect Arduino to the two temperature probes and two MOSFETs as shown in the electrical circuit schematics

9| Optional: Install two fans, connected to the main power supply, for air cooling within the control box

---

[8] http://smoothieware.org/troubleshooting#power-supply-problems

[9] http://smoothieware.org/stepper-motors

[10] http://smoothieware.org/endstops

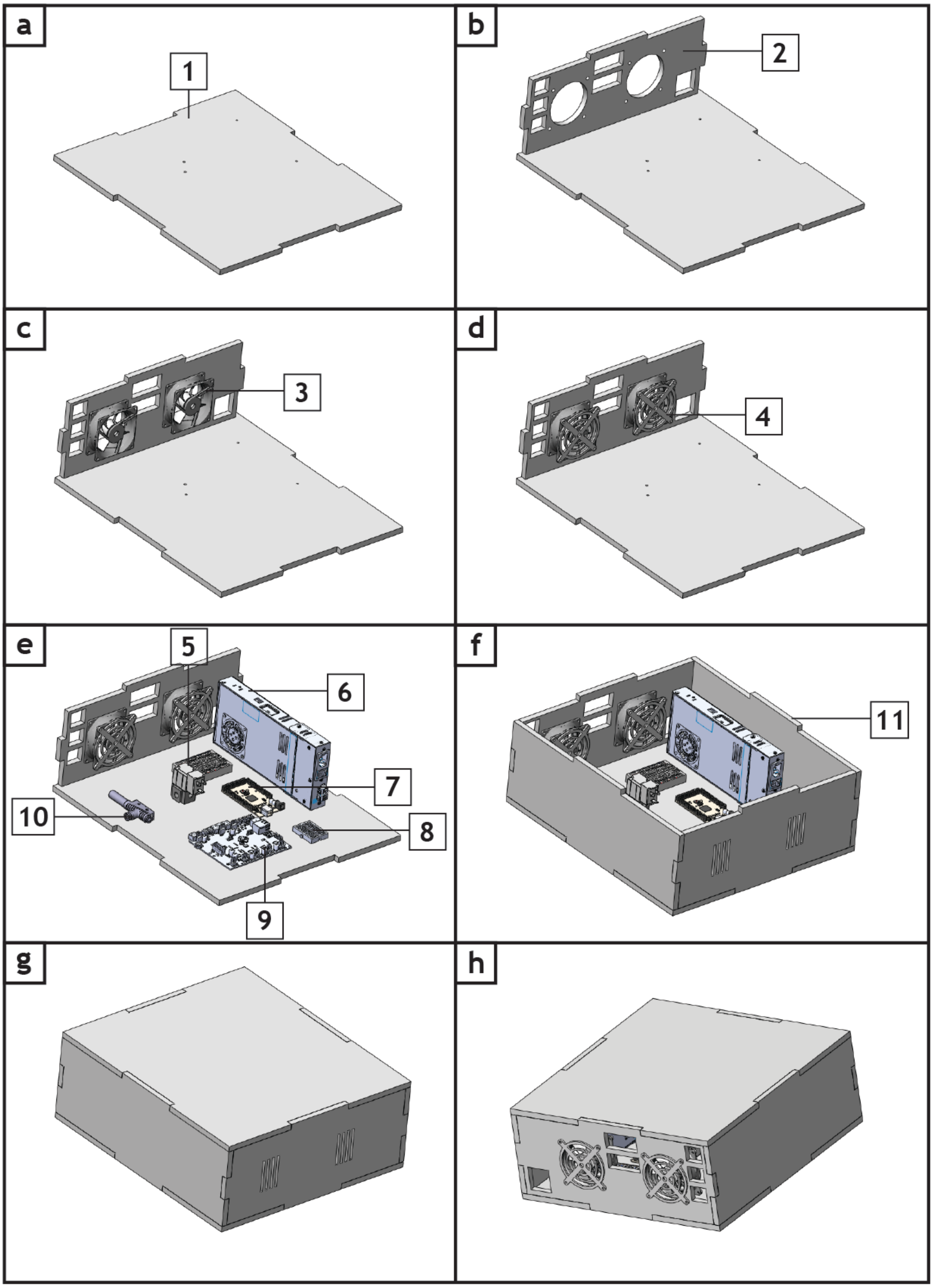

Figure 4: Assembly process for the control box (a-h): Parts: acrylic base plate (1), acrylic back wall (2) with fans (3) and fan grids (4), valve (5), power supply (6), Arduino MEGA, (7) bread board (8), smoothieboard (9), vacuum ejector (10), acrylic side walls (11).

6 List of tools

Please find below a general list of the basic tools required to setup the system:

- M3, M5, M6, M8 Allen keys
- Fine (or flat tip) soldering iron
- Tweezers
- Optional, optical microscope to inspect soldered connections
- Wire cutters
- Wire strippers
- Heating hose
- Heat gun
- Multimeter
- Pliers
- Meter stick
- Drilling machine with a drill set
- Thread cutting set with M3 and M5
