# Supplementary material for: OpenWorkstation: A modular open-source technology for automated in vitro workflows
Source: HardwareX. 2020 Oct 20;8:e00152. doi: 10.1016/j.ohx.2020.e00152 (PMC9041211; doi:10.1016/j.ohx.2020.e00152)
Supplement: Supplementary data 2 [file mmc2.pdf]

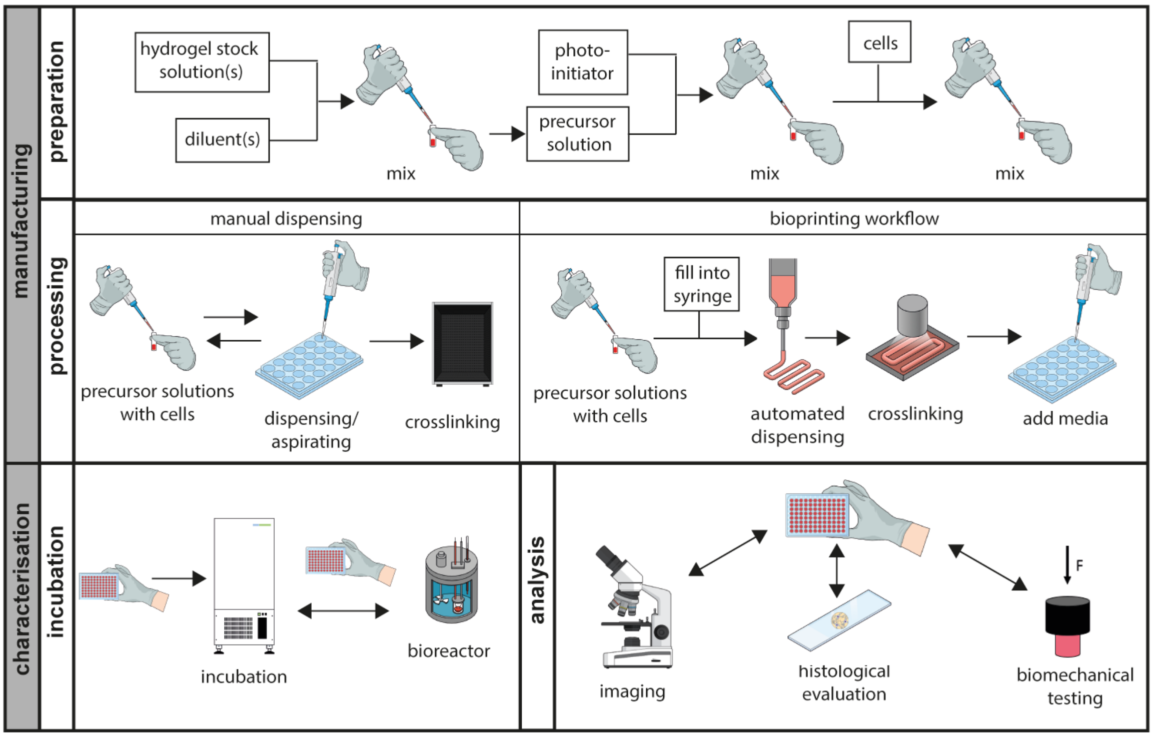

Supplementary Figure 1: Illustration of the current manual-based workflow for the manufacturing and characterization of hydrogels used for 3D cell culture. Reproduced with permission [17]. Copyright 2019, IOP Publishing.

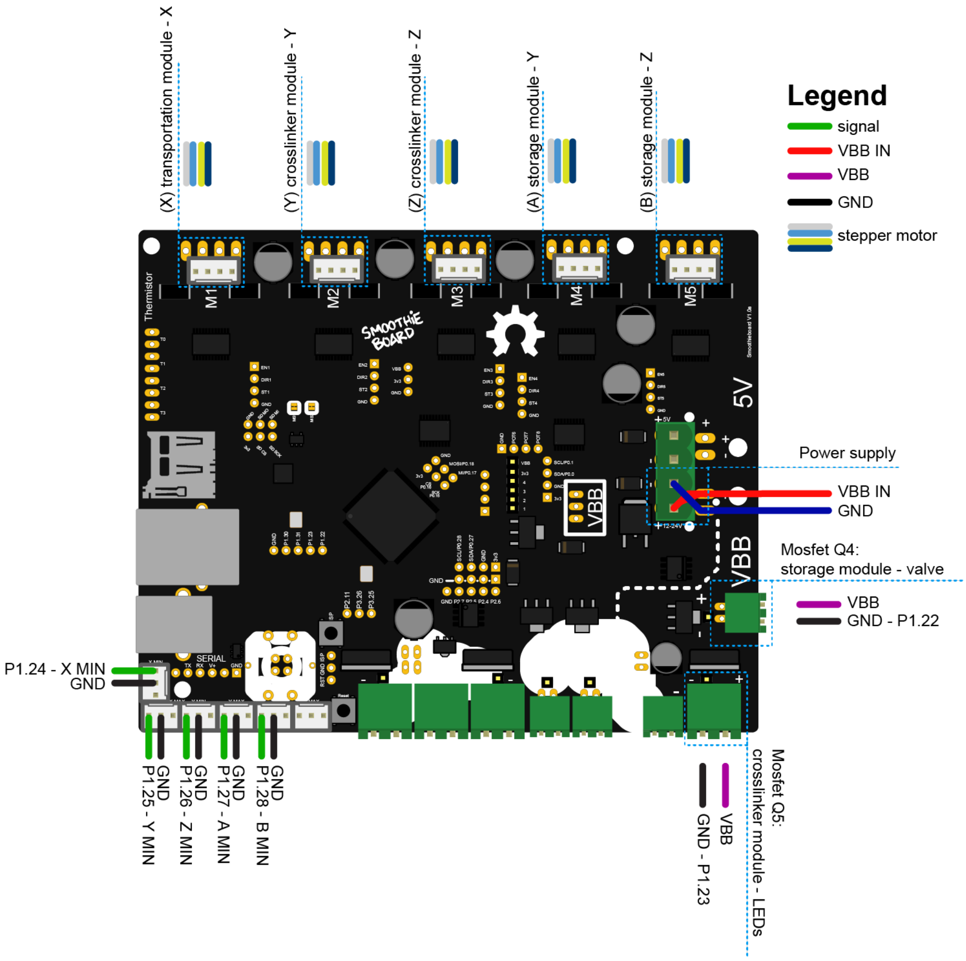

Supplementary Figure 2: Pin configuration of the smoothieboard. Detailed pin configuration including unused pins is available from http://smoothieware.org/pinout.

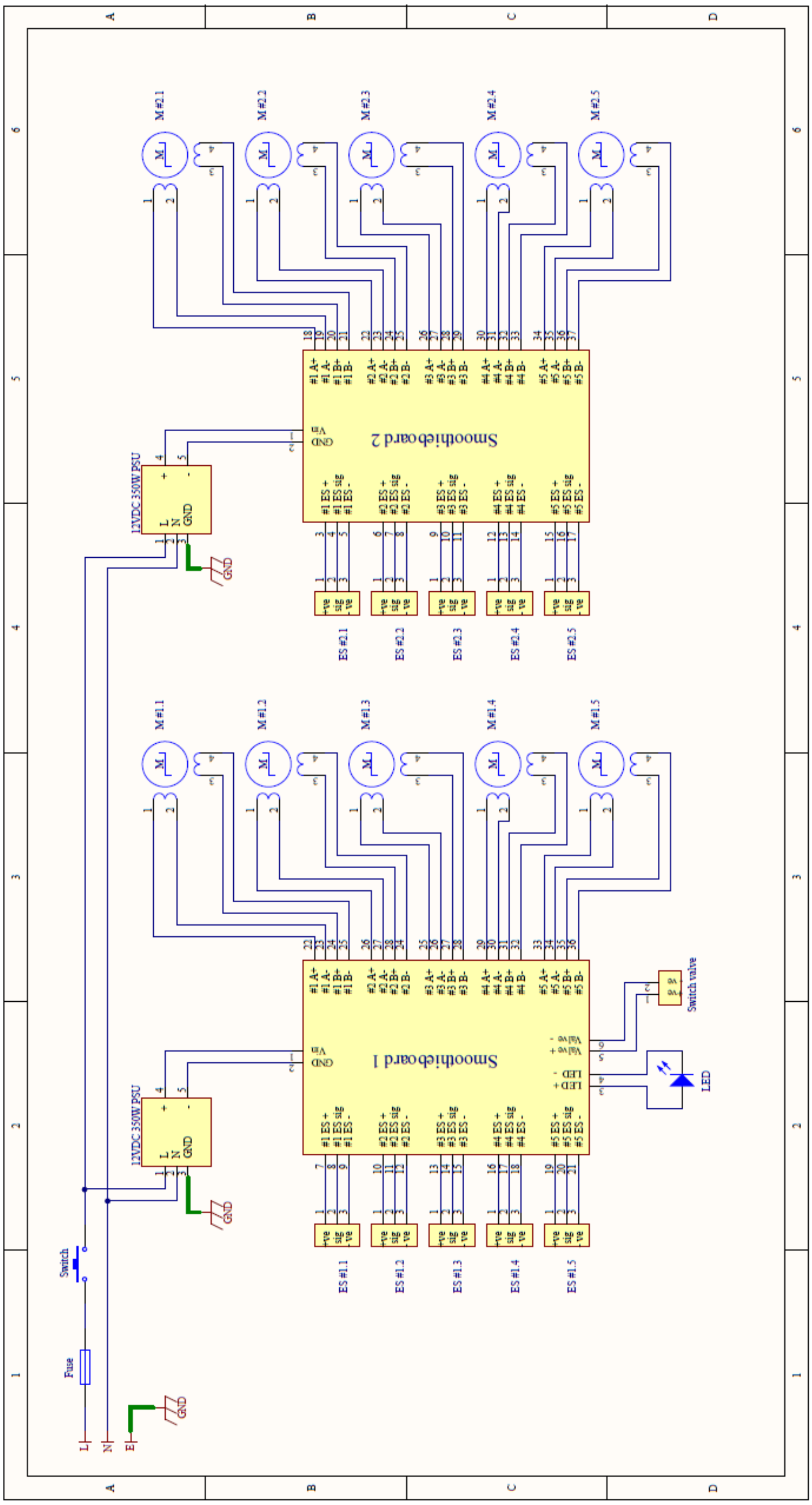

Supplementary Figure 3: Electrical circuit for the presented case study. A high quality and updated PDF is available on GitHub and includes a detailed description of the implemented parts.

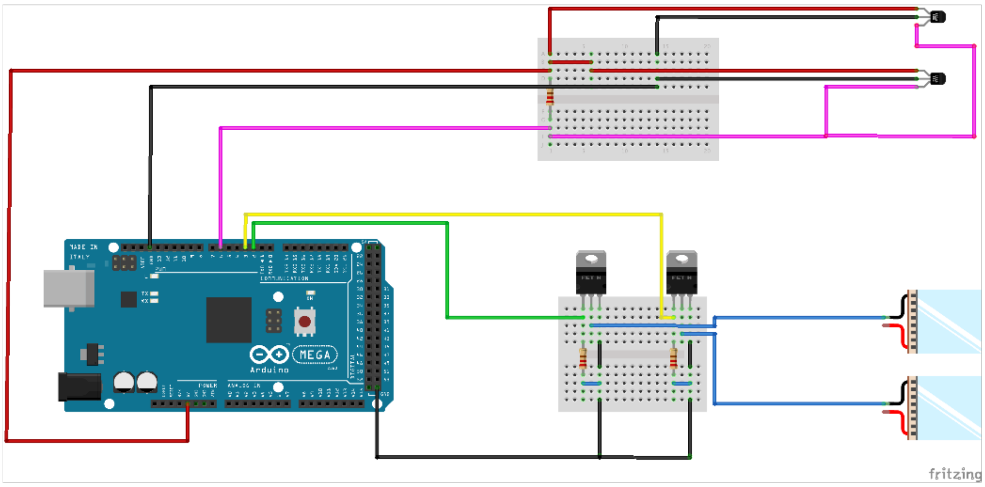

Supplementary Figure 4: Schematic of the temperature docks.
